# Innovative Bionics Product Life-Cycle Management Methodology Framework with Built-In Reverse Biomimetics: From Inception to Clinical Validation

**DOI:** 10.3390/biomimetics10030158

**Published:** 2025-03-03

**Authors:** Kazem Alemzadeh

**Affiliations:** School of Electrical, Electronic and Mechanical Engineering, (ESDI) Research Group, University of Bristol, Bristol BS8 1TR, UK; k.alemzadeh@bristol.ac.uk

**Keywords:** biomimetics, bionics, bio-inspired design, bionics PLM methodology framework, reverse engineering, technical biology, reverse biomimetics, bionic engineering, pictorial knowledge abstraction, nature design intents

## Abstract

This study uses bionics as an enabling methodology to bridge the gap between biology and engineering for generating innovative designs for implementation into novel technology development. A product lifecycle management (PLM) methodology framework is proposed that uses bionics as a technical discipline. The manuscript presents a novel, reverse biomimetics as a shape abstraction methodology to investigate, analyse, and de-feature biological structures through functional morphology as the enabling methodology for studying the relationships between form and function. The novel reverse engineering (RE) format with eleven stages supports technical biology, addressing the abstraction issues which have been identified as the most difficult steps in Fayemi’s eight-step framework. Inverse biomimetics and RE changes functional modelling (FM) from highly abstracted principles to low- or even reality-level abstraction, achieving nature design intents. The goal of the reverse biomimetic approach is to implement functional feature extraction, surface reconstruction, and solid modelling into five stages of a design process. The benefit of virtually mapping this in a pictorial fashion with high-end software fosters a simpler understanding and representation of knowledge transfer from biology to engineering, and can lead to innovative bio-inspired developments. The study aims to present the bionics PLM framework and its comprehensive processes of bionic design and biomimetic modelling, simulation, optimisation, and clinical validation techniques for two large-scale, human skeletal biological systems: a drug-releasing chewing robot and an anthropometric prosthetic hand suitable for introduction to engineering courses. Integration into undergraduate courses would be one route to bolster interest and encourage growth within the subject area in future.

## 1. Introduction

Biomimetics, bionics, and biomimicry are defined by the International Standards Organisation (ISO) 18458 2015 [1]. Biomimetics is an interdisciplinary cooperation of biology and technology (or other fields of innovation) with the goal of solving practical problems through the functional analysis of biological systems. In addition, this covers their abstraction into models, and transferral to a solution [2]. Bionics describes a technical discipline that seeks to replicate, increase, or replace biological functions by their electronic and/or mechanical equivalents. Biomimicry is defined as philosophies and interdisciplinary design approaches taking nature as inspiration to meet the challenges of sustainable social, environmental, and economic development [1,2]. There are three types: one is mimicking form and shape, the second is mimicking a process (e.g., photosynthesis), and the third is mimicking at an ecosystem level (such as planning a naturally inspired city) [3]. Generally, biomimetics applies principles and strategies from biological systems to engineering and technological products, processes, and designs [4], whereas bio-inspired design (BID) is defined as an approach that uses analogies to biological systems to extract innovative solutions to solve difficult or complex engineering problems [1,5]. In short, BID is a creative interdisciplinary process between biology and technology [6]. It is an emerging field of research with increasing achievements in engineering for design and problem solving, and its impact is significant for product innovation. Over the past two decades, biomimetics has established itself as one of the most promising strategies to support innovative [7] and environment-friendly products [8,9,10,11,12]. The innovation process in biomimetics or BID starts by linking a biological system to a specific technical question. The key feature of biomimetics is the unification of knowledge from the field of biology to obtain practical, technical implementations [1].

Comprehensive studies by several authors [4,9,10,13,14] have aided this innovation, and the recent review by Velivela and co-workers [15] developed methodologies to define the process of emulating biological principles or strategies through a sequence of steps. The procedure for executing such steps are described within a framework, and are accompanied by tools assist in performing the steps [4]. The recent review by Lenau and co-workers [16] explains the ISO model [1], Biomimicry Design Spiral [17], Georgia Tech model [18], Paris Tech model [19], DTU Bio-cards, and level of abstraction [20]. Although each framework differs in the number of steps, they follow a similar sequence starting from problem definition and finishing with evaluation. Frameworks such as the Biomimicry Design Spiral contain added value by using taxonomy to arrange biological systems, which aids in the selection of analogies. Lenau and co-workers also indicated that there are different versions of the description of existing frameworks, methods, and tools. Such descriptions were purely subjective, and depended upon the type of evaluation performed during the BID process. They highlighted that the eight-step process developed by Fayemi [14] represents a holistic and detailed sequence to perform a biomimetic process. Fayemi’s eight-step process model presented in Table 1.

Table 1 shows the process with its double symmetrical abstraction in step 2 and step 6, as highlighted. In step 2, the technical problem is abstracted from the initial analysis of the design problem. In step 6, key biological principles or strategies are abstracted from the understanding of a biological phenomenon and brought into a form useful for design work.

Prior to the development of this framework, Fayemi and co-workers [14] reviewed the terms and definitions for bionics, BID, biomimicry, and biomimetics from ISO/TC266 2015 [1] with respect to solution-based and problem-driven approaches [21]. Their review related the terms to bioinspiration for fostering creativity from mere inspiration up to novel design solutions. They distinguished the terms according to specificity, analogy, and an axis of related fields such as mechanics (bionics), sustainability (biomimicry), and other fields including nanotechnology, materials science, architecture, aerodynamics, or molecular engineering. Furthermore, they identified twelve biomimetic process models from 2004 to 2015 [1,5,18,21,22,23,24,25,26,27,28,29] aligned with problem-solving. They mapped and colour-coded these models with their specific features, and presented them in comparison with each other’s included number of steps. Their analysis outlined an eight-step process model in two phases, designed as a “double symmetrical abstraction-specification cycle”. The first phase (steps 1–4) focuses on a technology to biology transition known as the technology pull approach [1], whilst the second phase (steps 5–8) takes a reverse outlook—a (bottom-up) biology push approach [1]. They identified the required contributions from biologists and technologists with these two phases, where the problem analysis step is the initial entry point of the unified biomimetic process model. The study also analysed types of biomimetic tools and the similarities between the eight-step problem-driven and technology pull approaches, leading to a proposed “utility tree presentation” as a visual framework for the practical implementation of biomimetics.

In a study by Graeff and co-workers [9], Fayemi’s framework was used as a reference to review biomimetic processes. They highlighted that a lack of biological knowledge led engineers to face limitations; for instance, how to find, extract, and transfer biological models. Their study reviewed three strategies to deal with biological data: (a) databases of structured models [27,30,31]; (b) natural language web searches [32,33,34] along with artificial intelligence (AI) [34,35]; and (c) approaches based on highly abstracted principles [6,36]. Each strategy possesses advantages and drawbacks; however, irrespective of the approach, the sixth step (abstraction of biological strategies) has a crucial impact during biological data abstraction, and appears fundamental in all biology-centred steps [9]. This step focuses on understanding and interpreting biological systems in a coherent manner. They proposed integration of biological researchers into the framework to aid the knowledge transfer (during the sixth step), which requires a deep understanding of the chosen strategy [37] before progression to a lower level of abstraction can occur [38]. In a later study by Graeff and co-workers [39], a tool called “LINKAGE” was developed for sharing information between biologists and engineers, with comprehensive guidelines to improve their communications with graphical representations. Later, Graeff and co-workers further developed [40] the tool though its expansion into an online collaborative platform to assist interdisciplinary teamwork during the analysis and abstraction of technological problems and biological solutions, although extensive evaluation was not carried out.

Nagel and co-workers [41,42] analysed several sources of design methods: biology literature, engineering design theory, Biomimicry 3.8 Institute literature, and current BID innovation literature [42], where the last two are known as the spiral design method. This was performed to develop a categorical view of how knowledge is disseminated from biology to engineering. They proposed category analogies to represent a biological system as a practical tool to transfer knowledge at multiple levels of abstraction [42]. This addressed the issue of fixation, as well as offering a model that guides learning and transferral of biological knowledge to solve a problem. The seven proposed categories are divided into four physical (form, surface, architecture, material), and three non-physical (function, system, and process) characteristics. The work compared their proposed model with Chakrabarti and co-workers [23] and Mak and Shu’s work [43], where they also split the seven categories model into two levels of abstraction of high (system and function) and low (form, surface, architecture, material, process). The study concluded that this category model can be utilised independently as the design method, and may be applied to professional product design, research, and teaching purposes. However, in a recent review by Weidner and co-workers [44], multiple common issues with this model [42] were listed. This included selecting the wrong features from the analogue to implement, poorly abstracting biological principles (leading to misapplied analogies), and dealing with aspects of distance, modality of representation, and expertise. They mentioned that analogies between the source and the target were used to simplify describing aspects of a design problem.

Graeff and co-workers [40] confirmed that abstraction was the most difficult step during biological knowledge transfer, and Nigel and co-workers [45] believed that the integration of biological researchers into the framework is necessary. Their study extended that of Nigel and co-workers [41,42] with three additional abstraction approaches [10,46,47] that led to analogy categories. Graeff and co-workers reviewed six different abstraction approaches [40], and proposed four prisms to the process: what, how, why, and when and where They provided a comparison table for these six approaches with their corresponding prisms. This included the analogy category approach by Nagel and co-workers’ work, which was addressed in the following manner: what (form, surface, architecture, material), how (process), and why (system, function) [42]. Their study analysed levels of abstraction with reference to Fayemi’s eight-step process [14], and characterised the abstraction axis into four pictorial levels (reality, low, intermediate, high). A comprehensive descriptive information for abstraction levels was also given, aiding in how to implement the eight steps throughout a biomimetic process.

A recent study by Wanieck and co-workers [48] also supported Fayemi’s eight-step process, and they claimed that enhancing the use of the biomimetic process with a step-by-step technical standard would increase applications in the practice of technical design, building a bridge to common engineering procedures. This claim was supported by one of their earlier studies [49], in which they conducted a comprehensive review of the ISO standards and VDI guidelines on biomimetics from 2012 to 2019. It identified that the terminology, concepts, and methodology of ISO 18458 could be stricter [1], and it was not well-established as a recognised method for product development and engineering design, especially in industrial procedures. As a result, a new VDI Standard (VDI 6220 Part 2—Biomimetic Development Process; Products and Procedures) was developed and drafted in 2022 by an interdisciplinary expert panel from the fields of biology, engineering science, and industry, which linked biomimetics to classical processes [48]. In this new standard [50], the biomimicry design spiral is split into four sections with continuous transitions. Each section represents a phase in the biomimetic approach, and contains phase-specific activities. The phases are arrange (I), analyse (II), abstract (III), and apply (IV), and contain similarities with the eight-step processes developed by Fayemi and co-workers and Wanieck and co-workers [14,48]. The standard encompasses both the biology-push (solution-based) and the technology-push (problem-driven) processes, and is intended to be used in any product development process to establish biomimetic applications in the future [48].

**Figure 1:**
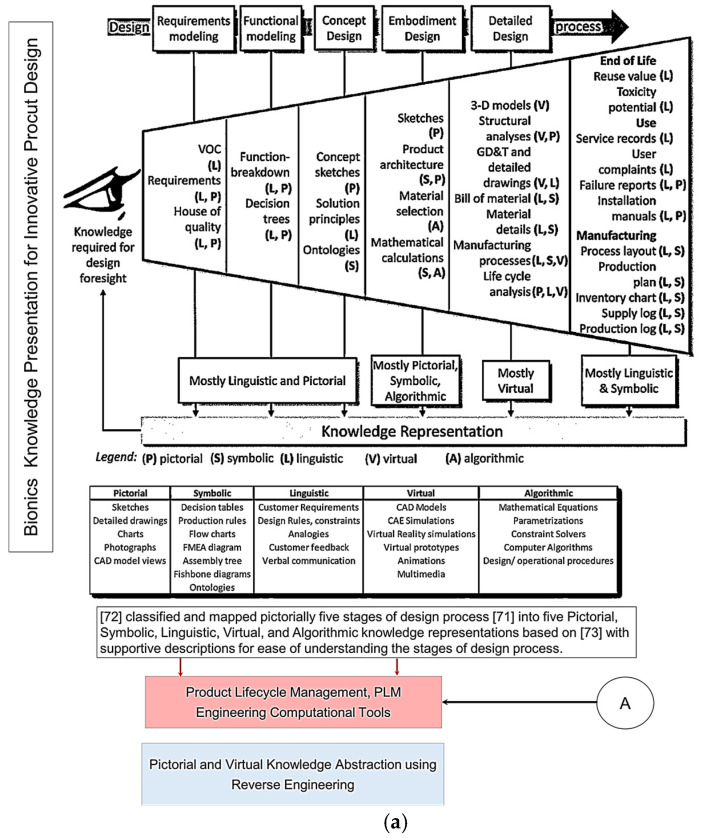

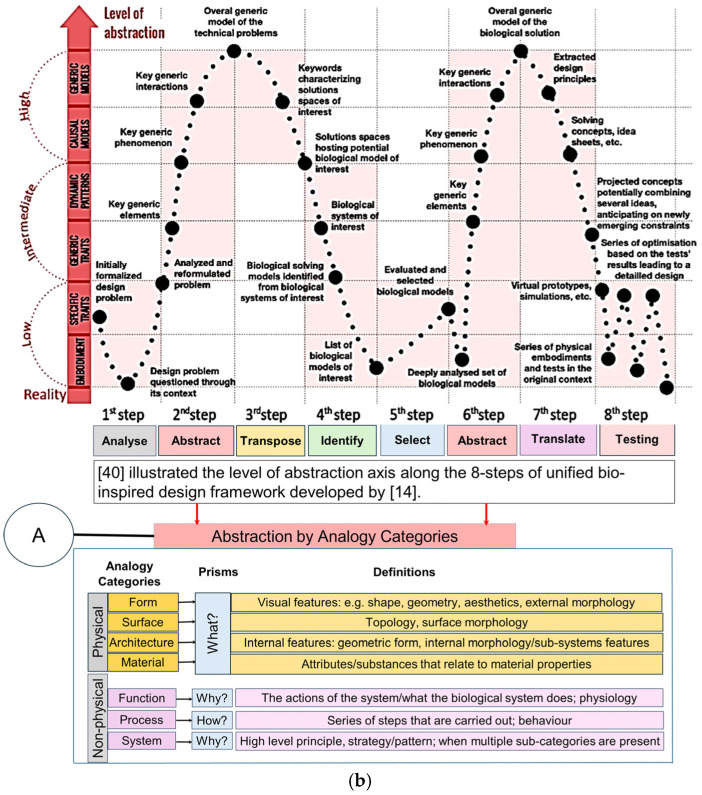
(**a**) Knowledge presentation framework for bionics based on knowledge abstraction through analogy categories based on VDI Standard 6220, Part 2. Adapted from [39,64]. (**b**) Knowledge presentation framework for bionics based on knowledge abstraction through analogy categories based on VDI Standard 6220, Part 2.

In this work, an innovative methodology framework is proposed; it uses analogy categories [41,42] supported by the four prisms of abstraction [40] to aid in identifying form-function connections. An innovative, biology-based reverse engineering approach (denoted as “reverse biomimetic”) is proposed as the abstraction method within the bionics’ framework; this is illustrated in Figure 1. The novel methodology utilises bionics as an enabling methodology to bridge the gap between biology and engineering for generating creative designs and implementing them into the development of novel/smart technologies [51]. Bionics is an interdisciplinary approach [50] combining life sciences with engineering sciences [52,53], and places an emphasis on functional modelling (FM) as the abstraction method [54]. The study shows that utilising bionics as the methodology for implementing technical biology towards naturally inspired solutions can generate innovative ideas [51] or “understanding nature with the means of technology” [27,55]. A novel reverse engineering (RE) [56,57,58,59] approach was proposed and presented in the context of product lifecycle management (PLM). RE is defined as the process of analysing a subject’s system in engineering or biology to identify its components and their relationships, as well as creating representations of the system in another form or at a higher level of abstraction. PLM is the set of processes and technologies used to manage the entire lifecycle of a product, from conception to disposal. The proposed RE approach with the PLM environment enables scientists and engineers to investigate, analyse, and transfer the underlying principles/mechanisms in nature throughout different stages of product development. The methodology addresses the abstraction (for definition see [50]) issues in two-phase, double symmetrical abstraction-specification cycles (steps 2 and 6), which have been identified as the most difficult steps in Fayemi’s eight-step process [14]. Moreover, this proposed framework supports both top-down (technology to biology) and bottom-up (biology to technology) approaches [60,61], which are aligned with Fayemi’s process [14]. It uses the modern VDI Standard 6220 Part 2, which provides a framework for the design of biomimetic products [50]. The study clearly shows unique examples of bionic engineering and large-scale synthetic biology, and how the technical biology is incorporated for creating the biological systems. The two examples are a set of humanoid jaws for a drug release chewing robot, and a hand’s skeletal structure for an anthropometric, tendon-driven prosthetic hand. They were conducted in the PLM environment, utilising Siemens NX CAD/CAM/CAE 2306 software for product development, where engineers can evaluate design intent, such as how the model behaves when dimensions are modified, or scaling the model with “what if scenarios” [62], or “contradiction analysis” [63]. This is the key engineering strategy adapted and implemented in high-end commercial PLM environment for product development.

Figure 1a top illustrates the five design process stages that Pahl and co-workers [65] proposed, which were subsequently chosen for the new VDI Standard [50]. Chandrasegaran and co-workers [64] classified and pictorially mapped [65] five stages of a design process into knowledge representations (pictorial, symbolic, linguistic, virtual, and algorithmic) based on Owen and Horváth’s work [66], with supportive descriptions for ease of understanding. Figure 1b illustrates the level of abstraction axis proposed by [39] along the 8 step of the unified bio-inspired design framework developed by [14,39,41,42,48,65,66]. Refs. [41,42] proposed seven category analogies based on analysis of four design methods to represent a biological system as a practical tool to transfer knowledge at multiple levels of abstraction. The seven categories proposed are divided into four physical (form, surface, architecture, material), and three non-physical (function, system, and process) characteristics. Ref. [39] proposed four prisms (what, how, Why, and when and where), and, as applied to seven category analogies approach [41,42], are what (form, surface, architecture, material), how (process), and why (system, function). Their study analysed the level of abstraction with reference to Fayemi’s 8-step process [14], and characterised the abstraction axis into four levels (reality, low, intermediate, high), and illustrated them pictorially for better understanding, as shown pictorially in the top.

The study aims to investigate the adapted reverse engineering (RE) approach [56,57,67,68] to implement the eleven stages of RE to support the technical biology and design intent for geometrical abstraction of biological structures. This aims to maintain the accuracy and integrity of form–function relationships and the processes between anatomical landmarks and biological structures. Moreover, the FM abstraction method implemented within the reverse biomimetic approach uses functional morphology as the enabling methodology for studying the relationships between form and function [69,70,71]. RE, with its eleven stages, changes FM from being highly abstracted principles [6,36] to low- or even reality-level abstractions, achieving nature design intents, as shown in Figure 1. The proposed approach uses geometric RE embedded with feature recognition and feature-based modelling to address the common issue of selecting wrong features from the biological analogue to implement [41,42,44].

Various applications within Siemens NX PLM 2306, specifically built-in tools for RE, were used to abstract different functional components (shapes, structures, patterns, textures, dynamics, behaviours, etc.) to reveal the constructional design and working principles. These tools, along with the comprehensive design, simulation, optimisation, and validation processes helped to realise the multi-skeleton modelling of a human skull and a hand. This demonstrates the cross-disciplinary (including clinical trials) processes using BID and biomimetics to enable researchers to visualise and analyse biological abstraction and knowledge transfer in the form of virtual and physical prototypes for innovative product development [72]. The study accurately illustrates how nature design intents can be captured through modelling functional biological features for shape reconstruction within a PLM environment [1,3]. This reverse biomimetic approach, adapted from Helfman and co-workers [70] and Speck and Speck [73], with associated industrial modelling tools and advanced manufacturing technology, aids in understanding both biological and technical systems, and is emphasised in two developed applications [60]: a humanoid drug release chewing robot [72], and a anthropometric, tendon-driven prosthetic hand.

The manuscript critically reviews the principles of bionics to support the novelty of the PLM methodology framework with built-in reverse biomimetics in Section 1.1, Section 1.2, Section 1.3, Section 1.4 and Section 1.5 (Figure 1). The implementation of RE with eleven stages, having six phases, are covered in Section 2.1 and Section 2.2 for a drug-releasing chewing robot and an anthropometric prosthetic hand, respectively. Clinical validations (Phase 6 of RE) of both applications are presented in Section 3.

### 1.1. Functional Modelling as an Abstraction Method

Figure 1 illustrates the five design process stages that Pahl and co-workers [65] proposed, which were subsequently chosen for the new VDI Standard [50]. It is a sequential model when compared with five other popular models: stage-gate, cyclical, double-diamond, V-models, and concurrent models. Chandrasegaran and co-workers [64] classified and pictorially mapped [65] five stages of a design process into knowledge representations (pictorial, symbolic, linguistic, virtual, and algorithmic), based on Owen and Horváth’s work [66], with supportive descriptions for ease of understanding.

Functional modelling (FM) [64] is described as an activity to develop models of devices, products, objects, and processes based on their functionalities and that of their subcomponents [74]. It provides an abstract, yet direct, method for understanding and representing an overall product or artefact function [75]. FM is the second step in the design process, and is a multi-disciplinary approach that guides designers during conceptual design [76] and supports in breaking down the overall function of the device into small, easily solved subfunctions—the configuration of the device follows from the assembly of all sub-function solutions [77]. Functional modelling is often considered a fundamental step in the engineering design process [64,78,79,80,81,82,83], which is presented in Figure 1. It is considered a useful diagram-based knowledge representation tool for modelling the functional design of biological systems when compared with other text-based or tabular tools. This is because functional decomposition can be central element of conceptual design: the third step concentrates on the embodiment phase, and the fifth step collates information into a detailed functional model of the design in question. In other words, physically deconstructing a product, process, or component for redesign (or curiosity), and analysing the interactions of the subfunctions, is a common method for creating a functional model [26,84].

Erden and co-workers [74] reviewed functional modelling, FM approaches and applications, and highlighted that FM bridges the gap between high-level requirements (the first step) and low-level details (the fifth step), as shown in Figure 1. Such a common model provides a holistic view of the system above the domains of different expertise, and makes it possible to review the design process and verify the satisfaction of high-level requirements through the lower-level specifications [74]. Eisenbart and co-workers [85] discussed the diversity of FM approaches across disciplines through different interpretations and definitions by researchers. The study analysed forty FM approaches in six different disciplines (mechanical engineering, electrical engineering, software development, service development, mechatronic system development, and product/service systems design). They suggested that there is no such shared sequence for FM, and proposed an integrated FM framework. In a later study, Eisenbart and co-workers [86] identified that the approach can be extended to interdisciplinary design [50], and for abstract modelling of biological systems [87]. Utilising functional modelling, FM as an abstraction method is popular in BID for knowledge transfer from biology to engineering to develop a new product because the scope or boundaries (i.e., customer needs and constraints) of the functional model are well defined by the physical models and/or modularity of those models [26,88]. In a comprehensive study by Wanieck and co-workers [4], FM was identified as one of the six categories to facilitate the process of biomimetics. They analysed forty-three biomimetic tools developed between 1987 and 2015, and categorised and classified them into six categories based on previous research by Fu and co-workers [13] and Fayemi and co-workers [14,19,89]. These were: AskNature [4,30,90], Biomimicry Taxonomy [91], Biologically Inspired Problem Solving, Biops [4], Ontology Explorer [92,93], Automatically populating the Biomimicry Taxonomy for scalable systematic biologically inspired design [94], and functional modelling (FM) [87]. They suggested that FM is a design modelling method for functional biological systems, where Fayemi and co-workers [14] referred to it as biological modelling. The study also defined ten variables for a qualitative (analysis, abstraction, application, transfer based) classification of tools based on Fu and co-workers [13] and Fayemi and co-workers [14,19,94]. The ten variables described the tools for FM were: $V_{1}:$ class = abstraction; $V_{2}:$ type = method; $V_{3}:$ step of process = six; $V_{4}:$ approach = both; $V_{5}:$ accessibility = open-source; $V_{6}:$ availability = print; $V_{7}:$ field of knowledge = biology; $V_{8}:$ dimension = needs previous step and facilities following step; $V_{9}:$ sustainability = no; $V_{10}:$ proof of concept = yes. The variables $V_{1},$ $V_{2}$, $V_{3},$ $V_{4},$ $V_{7}$, $V_{8}$, and $V_{10}$ indicate that FM is an open-source method with six-step for abstraction of biological models and a proof of concept of innovative products which can be solution-based or problem-driven based on the field of biology and technology, respectively.

The importance of function was highlighted in a recent study by McInerney and co-workers [6] where function was defined as a key central concept to the practice of biomimicry. Their study indicated that a functional approach would be a bridge between biology and engineering, enabling practitioners from a variety of backgrounds to more easily communicate and collaborate during a biomimicry design process. Moreover, facilitating user interaction is a key concept to the practice of biomimicry, and it has been identified as a solution to overcome the difficulties of its interdisciplinary nature. Similarly to Vincent [95], they suggested that analysis of function was necessary for more systematic understanding of the complex biological systems as trade-offs in biomimicry design. McInerney and co-workers [6] also identified two important tools (Biomimetic Ontology [96] and Engineering to Biology Thesaurus [97,98]) and emphasised that, if bridged, they could facilitate a more systematic approach to biomimicry. They proposed an integrated tool called Engineering to Biomimetic Ontology (E2BMO) based on Fayemi’s eight-step process [14], where the Engineering to Biology Thesaurus relates to steps 2 and 3, and Biomimetic Ontology relates to steps 2 to 7. The use of E2BMO has enabled practitioners to better interact with complex biological knowledge without a heavy investment of time and energy, encouraging a more widespread implementation of biomimicry. However, the study by McInerney and co-workers [6] clearly indicated that E2BMO tool did not conform to Fayemi’s eight-step process [14]. Step seven (transposition to Technology), which is shown in Figure 1, is bypassed in both approaches. This is identified as a challenging aspect of carrying out BID, and becomes even more difficult in the final commercialisation stage due to a lack of funding in transitioning technologies from laboratory to application [99]. More specifically, performing the steps required to overcome the Technology Readiness Level, referred to as the Valley of Death transition, which is the stage between Proof of Concept (PoC) and a startup company [100,101,102].

The latest study by Snell-Rood and Smirnoff [71] acknowledged the importance of function as an interdisciplinary bridge in BID that can allow engineers and designers to transition between biological models and human applications. They highlighted that abstracting a problem into general functions allows designers to look for traits that perform analogous functions in biological organisms. Their study used Fayemi’s eight-step process [14], and suggested supportive information for steps 2–5 to broaden the range of potential biological models in BID. They describe ‘design functions’ as part of abstracting a technical problem, used function as a bridge, broadly explored functions, and identified functional trade-offs. Their research also investigated the trade-offs for selecting several biological models and human applications to illustrate that the concept of function could be used as a bridge to biology.

Nagel and co-workers [26] used [45,87] a systematic approach towards BID. They utilised functional representation to abstract biological systems for concept generation in engineering design [103], and provided a set of guidelines for biological modelling [70] in a repeatable and systematic manner that can be paired with existing function-based design tools [26]. Their studies indicated that abstraction plays a major role in the early stages of engineering design, and is a valuable tool during the conceptual design phase [104]. However, FM [45,105] is an approach using highly abstracted principles [6,36] for knowledge transfer from biology to engineering. In a recent review study by Weidner and co-workers [44], a sketching technique was proposed to facilitate the translation of biological systems to technical design, bridging the knowledge transfer gap and addressing the issues of fixation in design and misapplied analogies. Sketching is identified as one of the knowledge representation techniques shown by Chandrasegaran and co-workers [64] (see Figure 1). The use of sketches and drawings in traditional engineering practices by design engineers allows for the manipulation of tacit knowledge between individuals [104]. Weidner and co-workers [44] argued that visuals such as photos and images can not only facilitate analogical representations to further knowledge transfer, but re-representation has shown to mitigate fixation [106,107,108]. It can also aid the designer in understanding form–function connections and facilitate knowledge transfer when the biological inspiration is obvious from visual inspection [42].

### 1.2. Reverse Engineering as Abstraction Method for Knowledge Transfer

Reverse engineering (RE) is defined as the process of analysing a subject’s system in engineering or biology to identify its components and their relationships, as well as creating representations of the system in another form or at a higher level of abstraction [109,110]. A wide range of applications have emerged from its utilisation in fields, including engineering and bio-manufacturing industries such as aerospace [111], medical sciences and bio-medicine [112], preserving cultural heritage [113,114], and disaster response [115].

In mechanical design, RE has been considered as a method to understand how a product works [83]. It is a process that can acquire design knowledge or geometric information from physical products by scanning/digitising an existing part, subassembly, or product into a 3D CAD model without engineering drawings [110,116,117,118,119]. It plays a critical role in reconstructing the original design of legacy parts for remanufacturing purposes [120]. The VDI 5620 Standard of RE geometrical data guides 3D data capture and processing, and for the selection of the correct technology [121].

More importantly, in biology, it has been critical in transforming natural objects such as bone [120] or teeth [122] into the digital world [119]. For the fundamentals of RE, refs. [56,57,58,59,67,68,119,123,124], a comprehensive overview of methodologies and performance evaluation [124], and a survey of current state-of-the-art digitisation/scanning techniques readers can refer to the literature by Marks [113], Geng and Bidanda [125], and Buonamici and co-workers [126].

Wilson and Rosen [127] proposed a detailed seven-step RE approach for idea and concept generation from biological systems, based on Pahl and co-workers’ design framework without considering FM [65]. They proposed steps to assist the designer in searching for solutions in nature with the goal of designing advanced engineering systems based on biological systems. Their study highlighted that it is a challenge for engineers without a background in biology to understand the abstraction of biological concepts to certain levels.

This research attempted to answer the same question that Wilson and Rosen [127] raised, and a challenge highlighted in Chandrasegaran and co-workers [64], by implementing FM into Pahl and co-workers’ design process [65] in combination with eleven technical RE stages [56,57,58,59,67,68] into a bionics’ framework (see Figure 1). The eleven stages are as follows:

(1) 3D data acquisition;

(2) Filtering and merging point clouds;

(3/4) Creating and re-pairing triangular meshes;

(5) Segmentation (partitioning into disjointed regions);

(6) Region/feature classification;

(7/8) Fitting primary (functional) and connecting surfaces;

(9) Optimising free-form surfaces to obtain design intent (including constrained fitting and surface fairing);

(10) Creating B-rep model (i.e., stitching surfaces and building up a topological structure);

(11) Creating solid model for CAD/CAM systems for downstream applications.

This bionic framework that incorporates RE stages is applied to Siemens NX software for product development. The platform enables engineers to evaluate design intent, such as how the model behaves when dimensions are modified, or scale the model with “what if scenarios” [62] or “contradiction analysis” [63] using different applications of the software. Every step in RE is carried out using a specific tool or function (e.g., segmentation, shape-fitting). The details of how these eleven technical stages are implemented are discussed in Section 2.1 and Section 2.2 for a drug release chewing robot and an anthropometric tendon-driven prosthetic hand, respectively.

It was previously mentioned that Figure 1 displayed a pictorially presented framework for bionics, which is an extension of other researchers’ works [14,40,41,42,48,64,65,66] showing steps 2 and 6 of abstraction. The innovative framework is aligned with Fayemi’s eight-step process [14], and supports analogy categories [41,42] for ease of knowledge transfer from biology to engineering. The four prisms help to identify different biological systems, and details for answering “how and why” questions about their form and function. RE tools embedded in the PLM software 2306 can be employed by the researcher to create extremely accurate, virtual CAD models based on form–function connections [42], changing the level of abstraction from high to low using functional feature-based modelling (i.e., bottom-up design) and assembling modelling (i.e., top-down design) to achieve the nature design intents.

### 1.3. Functional Feature-Based Modelling as a Shape Abstraction for Knowledge Transfer

Feature-based parametric modelling is widely applied in industry using CAD modelling to create product parts and assembly models [128,129,130,131]. Associate feature modelling was introduced by Ma and Tong [132] to bridge the gap between knowledge-oriented tools and CAD applications. Chen and Ma [133] extended this concept, and developed a functional feature-based modelling scheme that included the functional requirements and the concept design into the process.

Mingqiang and co-workers [134] conducted a survey of shape feature extraction techniques that described and compared 40 techniques. Feature-based modelling supported geometrical and non-geometrical feature associations, which included factors from the higher level knowledge model developed by Chandrasegaran and co-workers [64]. In addition, they suggested that it can be integrated into CAD modelling, where functional decomposition is applied to break down an overly abstract function into several primitive subfunctions [131]. A survey by Li and co-workers [131] also highlighted the importance of feature-based modelling. They introduced “abstract geometry” to provide an intermediate between abstract functions and concrete geometries [129,130] to capture the fundamental geometric elements of the design functionals. This can alleviate the issue of interoperability in PLM, and support application of features in emerging technologies, including Internet of Things (IoT), big data, social manufacturing, and additive manufacturing (AM).

Cheng and Ma [129,130] proposed a functional feature-based CAD modelling method to guide designers in building CAD models for effective representation and communication of the design intentions. They created a unified modelling language diagram to represent functional features as a standard way to visualise a system’s design. They used a top-down design as an assembly modelling approach that can drive multiple part designs by using a single ‘‘parent” part. Users would create geometries at the assembly level (the parent part), and then move or copy the geometry to one or more components (the child parts) for bottom-up design. In their proposed framework, they organised the functional relationships, and properly constrained and parameterised the design elements to abstract geometry features to satisfy design intentions. Furthermore, their study demonstrated a modelling procedure to abstract geometry features for a connecting rod using Siemens NX. Abstract geometry features were extracted and presented on the connecting rod model, but not during the modelling procedure. They used abstract geometry features such as references, geometric entities, parameters, and constraints/relations as functional concept carriers to provide a suitable form of geometry for conceptual design and guidance for modelling. They applied the technique to a crank-slider mechanism in a combustion engine and provided design abstraction and the embodiment of geometries on different levels [129,130].

The research herein focused on free-form modelling and feature extraction from 3D measured point clouds. This study uses the assembly modelling technique (i.e., top-down design) and a geometric feature-based modelling approach (i.e., bottom-up design) akin to previous work by Cheng and Ma [129,130], but for biological structures with extensive clinical and biological features embedded into part and assembly models. It uses a hybrid segmentation process (i.e., technical stages 5 and 6) [135,136,137] for free-form objects, where segmentation is defined as the process of partitioning a polygonal mesh into an accurate and consistent region structure [56,58] and biological features used for shape abstraction. Moreover, the method is capable of functional decomposition of a 3D shape to discover the design intentions of scanned 3D point clouds [59], where shape abstraction of functional features for knowledge transfer are clearly demonstrated. There are generally four categories for 3D shape identification and segmentation of geometric primitives in point clouds: edge-based methods, region-based methods, clustering-based methods, and model-based methods [135,136,137]. Region/cluster based methods use mean or Gaussian curvature as indicators. Clustering is identified as the basis of shape segmentation methods based on the Gaussian map [135] for RE, where shape clustering enables the segmentation or partitioning a shape into subsets (clusters), such that objects in a cluster are grouped based only on information extracted from the data/point clouds that describes the shape [110]. The hybrid method is capable of functional decomposition of the 3D shape to discover the design intent of scanned 3D point clouds to support FM [56].

Hybrid region-based classification methods [138,139] were used in this manuscript as shown in Table 2 and Table 3 for complex free-form and biological structures. In Table 2, the principal curvatures are combined to give useful measures of the curvature of the surface, the HK (Mean-Gaussian) uses the signs of H and K curvatures in combination as two shape indicators to characterise surface shapes. In Table 2, SC (Shape Index and Curvedness), S uses a number in the range [−1, 1] with its associated colour map. Conditions (S = (0, ±0.5, ±1)) are critical points (CPs) when shape classification changes. C is intensity of surface curvatures.

### 1.4. “Reverse Biomimetics”—Technical, Biology-Based Reverse Engineering as a Shape Abstraction

The goal of RE frameworks are to extract functional features and reconstruct surfaces [140,141] (i.e., functionally decomposed surfaces) from 3D point clouds. This is to recreate a final, 3D digital free-form model that meets the design intentions [56,57,58,59,67,68,113,119,120,121,122,123,124,125,126]. This may be in the form of a 3D mesh or parametric 3D CAD model. Commercially dedicated RE software (NX Imageware, Geomagic Design X, etc.) can be useful for specific tasks; however, they are not capable of investigating the relationships between form and function, or morphology and function, to identify geometric relationships and carry out “contradiction analysis” or “what if scenarios” to optimise the intended product development [62,63].

In this study, Siemens NX and NX Imageware were used due to their extensive capabilities and built-in tools. Shape abstraction was demonstrated through the eleven technical RE stages and applied to an innovative drug release chewing robot and anthropometric tendon-driven prosthetic hand, respectively. The details of the eleven stages are covered in the five phases in Section 2.1 and Section 2.2. Moreover, Siemens NX allows users to carry out multi-body dynamics, FEA, prototyping and testing, changing the level of abstraction from high to low, and developing real-world solutions, as illustrated in the two examples of large-scale human skeletal biological systems [142].

### 1.5. Large-Scale Human Skeletal Biological Systems—Skull and Hand

#### 1.5.1. Strategies for Capturing the Nature Design Intents

The first technical stage (3D data acquisition) is one of the most important stages in the bionics framework. Two different strategies were adopted due to the nature of the skull and hand complexity. In this study, two SOMSO skeleton models of a human skull [143] and a hand [144] (SOMSOMODELLE GmbH, Adam Rouilly, Kent, UK), were used to create virtual biomimetic models. These anatomical models were chosen because their geometrical information and anatomical landmarks represent on these anatomical models passed scientific accuracy test. Two different scanning systems were used: touch-trigger probe or contact, and optical/non-contact. Touch-trigger probes were used to directly measure the skull, in particular tooth morphology to obtain the complex free-form occlusal surfaces information such as tooth morphology and dentition occlusal surfaces with associated traits. Two different non-contact scanners were used to indirectly measure the hand due to the number of parts (29), the complexity of free-form shapes, and pre-alignment/registration.

For a skull, a Renishaw Cyclone™ contact scanner (Renishaw plc, New Mills Wotton-under-Edge Gloucestershire, UK), Renishaw plc, New Mills Wotton-under-Edge Gloucestershire, UK with the associated software was used [145].

For a hand, combinations of non-contact, 3D white light scanners from Solutionix Rexcan 2/4 (Solutionix, Seoul, Republic of Korea) were used for digitisation processes. Both scanners were using white light scanning method. They used a direct video feed from twin-coupled cameras to capture images of multiple known patterns of white light that is projected onto the object that is to be digitised. These patterns were interpreted by Solutionix ezScan™ 2017 software using referencing and changes in the shape of the known pattern to produce point clouds. Rexcan 4 was first used to scan the hand to maintain the global coordinate system, then individual bones were scanned with dental Rexcan 2, which is ideal for scanning small bones. Rexcan 4 was also used for the larger bones (for example, proximal phalanges and metacarpal bones), as it possesses a larger chamber. Stratasys FDM and Ultimaker with their associated software were used for prototyping bones and passive extensors using PLA and Ninja semi-Flex materials, respectively.

#### 1.5.2. Skull 3D Data Acquisition/Digitisation

The study of bones and muscles, in particular dentition and occlusal surfaces, were necessary prior to digitisation to understand functional morphology of the bones and the mechanism of chewing [72]. This biological information and technical abstraction prior to the digitisation process is key to successfully extracting nature design intentions and associated features (i.e., ‘design functions’) as part of abstracting a technical problem [71]. Necessary arrangement of the 3D scanners and fixtures was carried out to find their capabilities for holding the objects to optimise the technical stages of the RE framework. In medical RE, specifically cranio-maxilofacial applications such as human skulls, biological shape analysis tools like 3D geometric morphometric analysis (GMA), and the use of referential geometrical entities (RGEs), are integrated to into RE process to determine the anatomical landmarks, maintaining the integrity of the processes between anatomical landmarks by representing the correct and clinically aligned biological structures, and also making sure that the reconstruction of the mandibular condyle and temporomandibular joint (TMJ) meet the correct restoration of articulation, occlusion, and mastication from a functional aspect, as well as the correct shape of the mandible or skull from the aesthetic point of view. The main advantages of integrating GMA or RGE methods are the powerful interpretation (size and shape) and visualisation of the results using principal and Gaussian curvatures to determine points, directions, planes of bone geometry, and the kinematic centres of condyles [72].

#### 1.5.3. Hand 3D Data Acquisition/Digitisation, RGEs and Segmentation Strategies

The anthropometric tendon-driven design of a prosthetic hand required understanding the structure of hand bones and their function. Joints, tendons, and muscles also play key roles in hand mechanics and dynamics and were also studied as a result. Shapes play an important role in the hand bones, determining the characteristics of the hand and dexterity. A human hand consists of 27, bones which can be divided into three groups: carpals, metacarpals, and phalanges. Carpals comprise 8 bones that are located at the wrist; metacarpals comprise 5 bones that are located in the palm; and, finally, phalanges are the 14 bones that form fingers. The eight bones in the wrist are organised into two rows: proximal and distal. The proximal row of carpal bones are the scaphoid, lunate, triquetrum, and pisiform bones. The distal row includes the trapezium, trapezoid, capitate, and hamate bones. (British Association of Hand Therapists (BAHT) anatomy handout [146]). The radius and ulna (i.e., the base of forearm) are very critical to digitise, as they act as references for pre-alignment/registration the bones of the hand. Together, they serve as the primary support structure of the forearm, articulating with the humerus and carpal bones. They also serve as origins and insertions for muscles responsible for flexion and extension of the forearm, wrist, and fingers [147].

##### Hand RGEs and Segmentation Strategies

**Figure 2:**
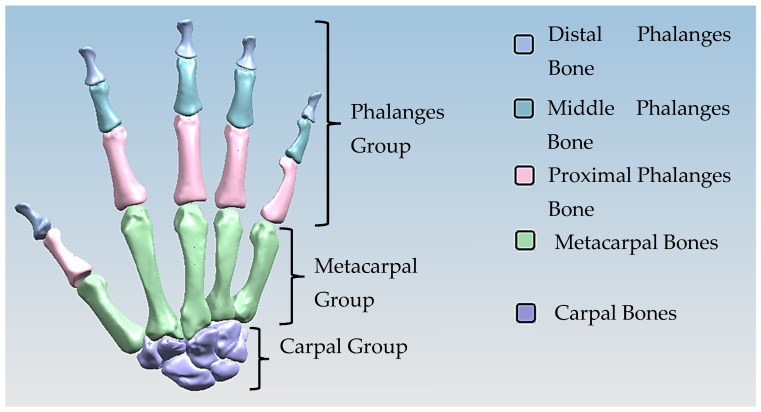
Three colour coded groups of hand bones for facilitating RGEs and the segmentation process.

Hand bones were further divided into three groups according to their shapes [148] for facilitating RGEs and segmentation process (see hand phase 3) as illustrated in Figure 2.

#### 1.5.4. Hand Evaluation Strategy—Synergy-Based Approach

Researchers have used the applications of hand synergies for novel design and control concepts of robotic hands and prostheses [149]. Synergy-based approaches have also aided in the development of superior robotic hands. Many synergy-based studies in the literature have used simple postures and grasps as part of their experiment [150]. Hand kinematic synergy extraction is widely applied in research to study human grasps, hand prosthesis control, gesture recognition, and for general rehabilitation [151]. In basic terms, a synergy defines a relationship between different joint angles in the hand, and can be used to control an entire hand movement by a single variable [152]. By designing the optimal shape and geometry (i.e., anthropometric) of a finger, even an underactuated hand can possess a stable grasp for a range of objects [153,154].

There are three types of synergy: postural, muscular, and neural [150,151]. Furthermore, the use of synergistic motions is a very promising approach to control high degree-of-freedom (DoF) devices that includes anthropometric hands [152]. “Ability to grasp”, and “ability to hold” are two distinctive functional features of cable/tendon-pulley driven hands [155]. Postural synergies are used for showing the changes in hand posture during motions such as reaching, grasping, and pinching objects with varying width, curvature, and angle. They represent basic building blocks underlying natural human hand motion [155]. The human hand has 27 DoFs [156]: 4 in each finger, with 3 for extension and flexion, and 1 for abduction and adduction. The thumb is more complicated, and has five DoFs. The remaining six DoFs are for the rotation and translation of the wrist.

Cobos and co-workers [157,158] analysed the kinematic behaviour of simplified human hand models to obtain the minimum and optimal DoFs for achieving efficient manipulation for power and precision grasps. They analysed and illustrated that grasping implies a strong relation among finger joints [158] by identifying that 9 to 14 DoFs are more precise for both types of grasps. A higher level of realism and sensitivity were achieved with models from 15 to 24 DoFs. With 15, 16, and 17 DoFs, it is possible to have the three important flexions for index, thumb, and middle fingers [157]. Only the model with 24 DoFs could perform simulations with an arched palm.

Inverse or forward-dynamic modelling (or combinations of both) are used for power and precision grasps and other computer-based musculoskeletal modelling [159,160]. The former uses physics-based models [160,161,162,163] that computes the motions that result from a set of muscle excitation patterns [160]. There are two types of forward-dynamic modelling: muscle-driven models and torque-driven models. Muscle-driven models incorporate effects of each muscle individually using the muscle–tendon complex representation. They have been widely used to investigate the function and contributions of individual muscles to different movements, yet their most frequently cited limitation is the difficult selection of realistic individual muscle parameters [164]. Torque-driven models use torque generators to apply the net effect of all muscles acting across a joint using a rotational muscle–tendon complex [164]. In this context, the anatomical joint laxity due to ligaments and synovial capsules can be characterised by a linear torsional spring and torsional damper applied at each articulation of the model [159]. This torsional spring-damper guides joint movement [163,164], and constrains it within the allowed physical angular limits, assuming that stiffness and damping components to each articulation and limiting all angular ranges of motion [159,165] are accurately specified.

Each model contains four stages: (1) model construction; (2) parameter determination; (3) model evaluation; and (4) model application. The first three stages are often an iterative process until the model incorporates sufficient complexity to adequately represent the real physical system [164]. Stage 3 is one of the essential steps in developing a simulation model before any application. McErlain-Naylor and co-workers [164] reviewed these four stages of development and the application of forward-dynamic simulation models for modelling sporting movements and highlighted that evaluation is an essential step in the process of developing a realistic simulation.

**Identification of hand centre of joints.**

##### Function of Joints

The metacarpophalangeal (MCP) joint connects the head of each metacarpal bone to the base of the corresponding proximal phalanx. They are grouped as condyloid joints, and can be considered to have two DoFs, namely flexion/extension and adduction/abduction. The centre of rotation (COR) of the MCP joint is located within the metacarpal head. This is true for the corresponding joints for proximal phalanges heads (PIJ or PIP), and middle phalanges heads (DIJ or DIP), and expect for the thumb, the interphalangeal (IP) joint is in the proximal phalange head and distal phalanges. The PIP, DIP, and IP joints are considered to behave like synovial hinge joints, which typically allow one DoF through flexion/extension.

The carpometacarpal (CMC) joint attaches the trapezium to the base of the metacarpal in thumb bone 9. It provides a wide range of motion, but can typically be categorised as a saddle type, two-DoF synovial joint. The osseous anatomy of the joint allows motion in three planes, whilst only directly having two axes of control [166]. The location of the COR dictates the possible range of motion (ROM) for the power and precision grasp [167] and the ability of the tendon and muscles to work in tandem [168]. ROM is often used as an indicator of successful hand rehabilitation [169]. However, the exact location of the CORs for any of these joints are not known, and they play a key role in functioning the corresponding joints in each fingers [168,169] to realise a realistic dynamic modelling of anthropometric human hand. The innovative integrated GMA and RGEs methods built-in the RE framework presented were applied to identify hand bones CORs, as explained next.

The Kanpandji test is a useful tool to assess opposition from the thumb and involves no external tools or implements [170]. The test is carried out by asking the patient to touch various points on their fingers with their thumb moving from position 0 to position 10. A score from 0 to 10 is then assigned depending on which positions can be reached. If all stages can be passed (a score of 10), then the thumb function can be considered normal. This test is vital to evaluate the effectiveness of prosthetics as thumb opposition is one of the most important functions of the hand.

## 2. Materials and Methods

Siemens NX and NX Imageware PLM software were used in combination to show how these RE tools are largely employed to capture biological design intentions. The methods are built-into five different approaches leading to different digital representations: (1) polygonal meshes, (2) quadrilateral patch layouts by automatic surfacing, (3) manually segmented surfaces, (4) functionally decomposed surface models, and (5) CAD models redesigned over meshes [58,59]. The (3)–(5) approaches can achieve design intentions and obtain high quality surfaces in the final model. Manual approaches can achieve higher accuracy other methods, but naturally are more time consuming. Várady and co-workers [58,59] proposed Morse segmentation (which is akin to hybrid methods) to create topological surface structures for feature free-form objects. The processes of segmentation, feature extraction and biomimetic modelling are extensively described and illustrated for two biological models (skull and hand) in Section 2.1 and Section 2.2, respectively. Simcenter 3D solver was used for the computational evaluation of hand bones form-function connections, validating the physical properties, mass and length, and also centres of flexion-extension rotation at the metacarpophalangeal and interphalangeal joints and the axes of flexion-extension and abduction-adduction at the thumb joint. A cartesian system of generalised coordinates and Euler parameters to solve the equations of motions were used in Simcenter 3D solver.

It was not the intention of this research to analyse each of these methods and extend the algorithms, which are well established and built-in to commercial software. The reader may refer to work by Theologou and co-workers [124] (a comprehensive overview of methodologies and performance evaluation frameworks in 3D mesh segmentation), Buonamici and co-workers [126] (reverse engineering modelling methods and tools: a survey), Di Angelo and Di Stefano [135] (geometric segmentation of 3D scanned surfaces), and Answer and Mathieu [110] (from reverse engineering to shape engineering in mechanical design).

### 2.1. Skull 3D Data Acquisition, Biomimetic Modelling

This section covers the eleven technical stages of the RE framework described in Section 1.2 which is split into six phases. The six phases consist of:

**Phase 1**—Digitisation Process.

**Phases 2 + 3**—Segmentation, feature extraction, and curve, surface and solid modelling.

**Phases 4 + 5**—Mapping clinical chewing trajectory and computational validation.

**Phase 6**—Prototyping, proof of concept, and clinical validation (will be explained in Section 3.1).

#### 2.1.1. Phase 1—Digitisation Process

**Figure 3:**
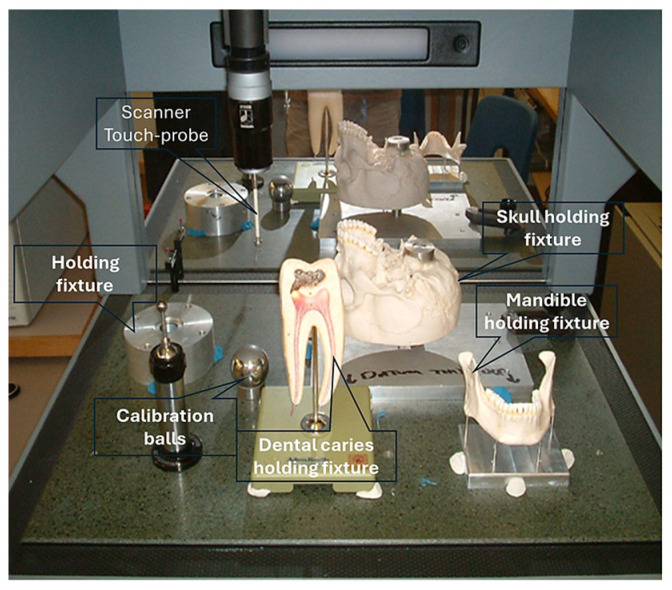
The Renishaw Cyclone Series II scanning machine with different holding fixtures with calibration balls used for biomimetic modelling of a human skull [69,171].

**Figure 4:**
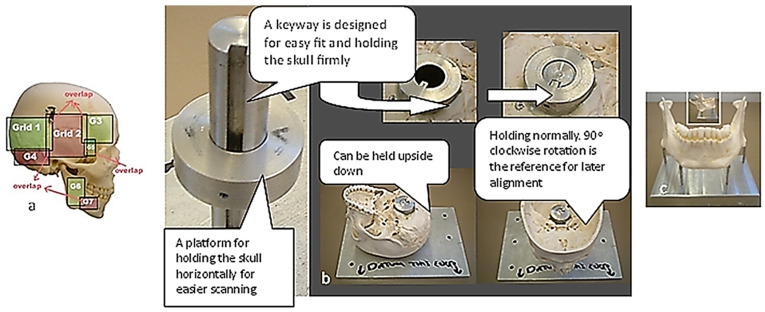
(**a**) Anatomical analysis of a skull structure from different angles to help grid design for planning digitisation strategy. The green highlighted regions represent the simple areas, whilst pink represents the critical and more complex areas. (**b**,**c**) Design of special fixtures to access anatomical features on the skull during the digitisation process. A good fixture design aided in scanning data without re-alignment [69,171].

**Figure 5:**
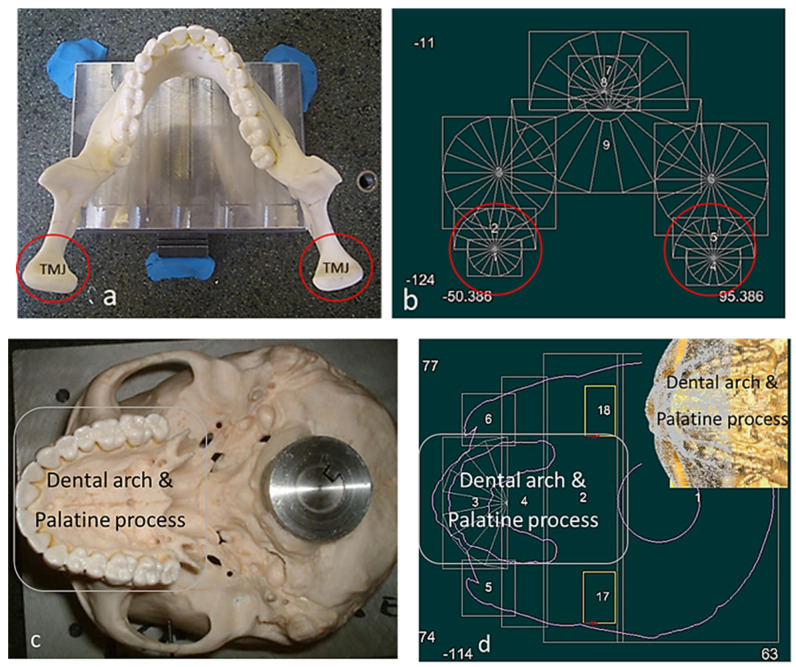
Skull structure planning and digitisation process. (**a**) Top view of mandible fixture. (**b**) Bionic grids design for the mandible (TMJ and teeth). (**c**) Bottom view of maxilla fixture. (**d**) Bionic grids design for the maxilla (dental arch, palatine process). Combinations of scanning techniques are used for digitisation processes, such as parallel to the X or the Y axis, angular or radial with different type of styli [69].

**Figure 6:**
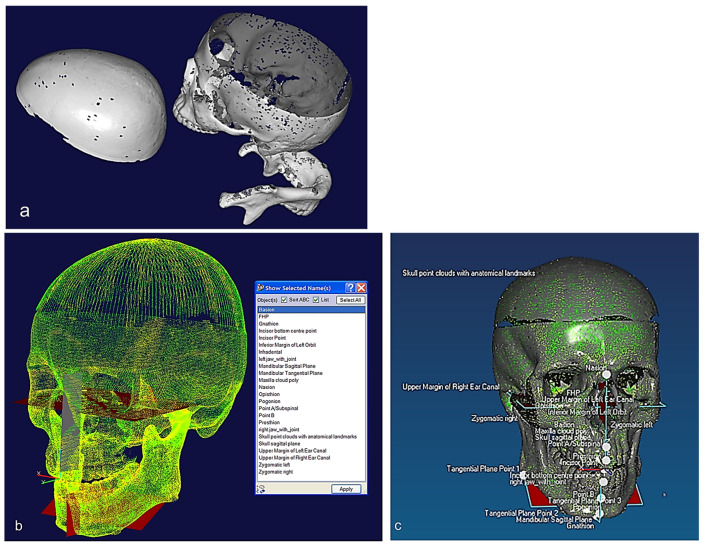
(**a**) Unregistered digitisation results of the skull’s three parts. (**b**) Point cloud of the skull with reference to global planes with landmarks identification. It shows three anatomical planes (i.e., the sagittal plane, the Frankfurt plane and the coronal plane), which divide the skull’s point clouds with cloud curvature colours, making it easier to visualise areas of high and low curvature and to locate features for feature extraction operations with proper neighbourhood size. (**c**) Polygonised point clouds with anatomical landmarks aligned to global reference planes using basion, nasion, and sella point on the maxilla, and the infradental, gnathion, and point B on mandible. The use of name, group, and layer with correct anatomical features have been used as shown in (**b**,**c**) [69,171].

This section covers stages 1–4 of the RE framework (see Section 1.2) for digitising a physical skull consisting of 3 parts (maxilla, mandible, and calvarium). The Renishaw Cyclone™ contact scanner was used [145] as shown in Figure 3. The process of designing bionic grids and the results of digitalisation (i.e., point clouds) for the mandible and maxilla are shown in Figure 4, Figure 5 and Figure 6.

#### 2.1.2. Phases 2, 3—Segmentations, Features Extraction, Curve, Surface, and Solid Modelling

**Figure 7:**
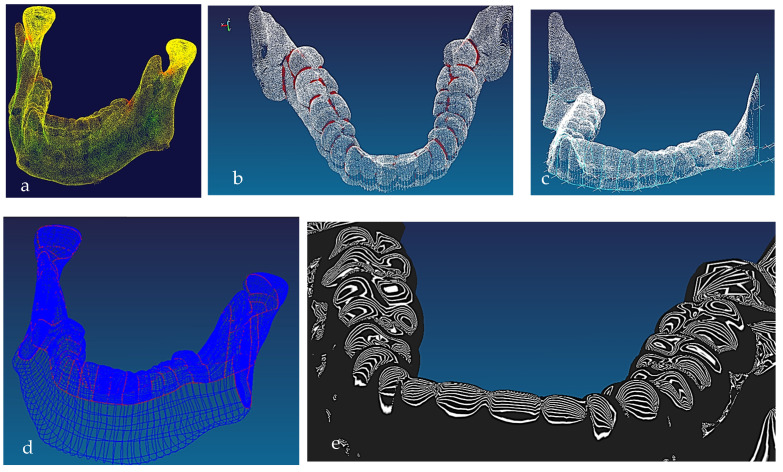
(**a**) Mandible point clouds and colours curvature analysis, (**b**) teeth threshold edge detection, (**c**) teeth point clouds and associated 3D B-spline curves network, (**d**) mandible parameterised curves network, (**e**) Zeba plot and surface quality analysis [69,171].

**Figure 8:**
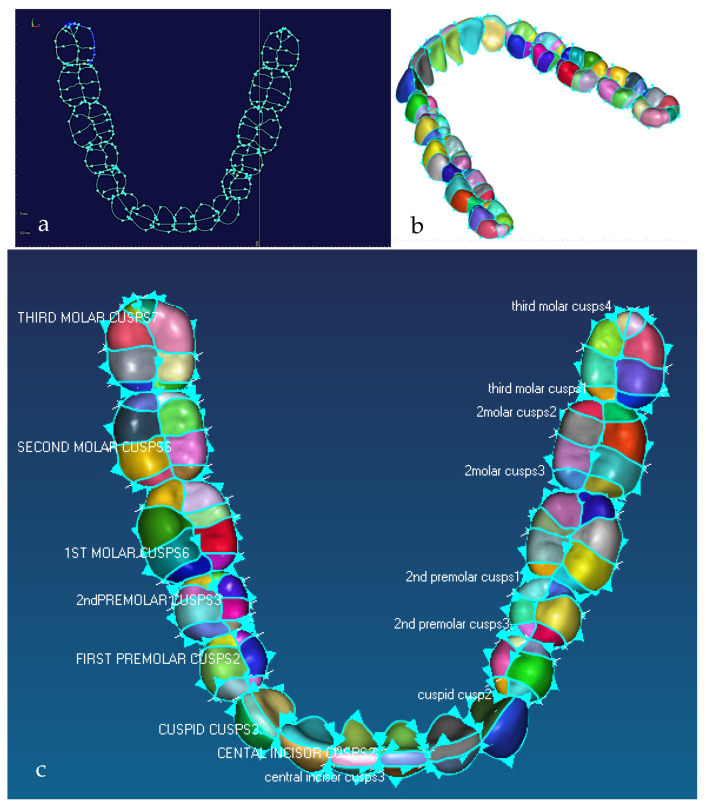
Illustration of the natural division of teeth into a number of cusps driven by the biomimetic modelling strategy, where 3rd molar is known as the wisdom tooth. (**a**) Detail of 3D feature extraction and identification of morphological traits of the mandibular teeth, with each cusp boundary curves and curve direction. (**b**) Tooth surfaces where teeth are naturally segmented into a number of cusps. (**c**) Details of mandibular teeth with their corresponding names and colour coded occlusal surfaces where 1st, 2nd and 3rd molar or wisdom tooth each has 8 cusps; 1st and 2nd premolar each has 6 cusps; and central and lateral incisor and cuspid has 2 or 3 cusps [171].

**Figure 9:**
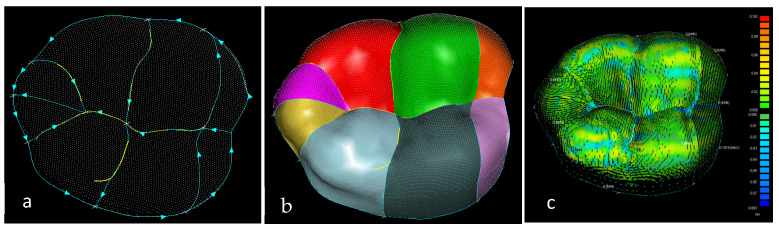
Detail of 3D feature extraction (i.e., identification of the morphological traits of the crowns) and 3D geometric morphometric processes, analysis and quantification of a 3rd molar having 8 cusps. (**a**) Morphological traits of the molar crown and B-spline curve network. (**b**) Cusps feature surface model. (**c**) Optimum tooth/cusp morphology, surface quality analysis with needle plot. The plot displays values of error which is the difference between the cusps surfaces created with the corresponding point clouds—the green colour on 8 cusps means the results are accurate (the maximum and average values are 101 µm and 7.5 µm, respectively) [171].

**Figure 10:**
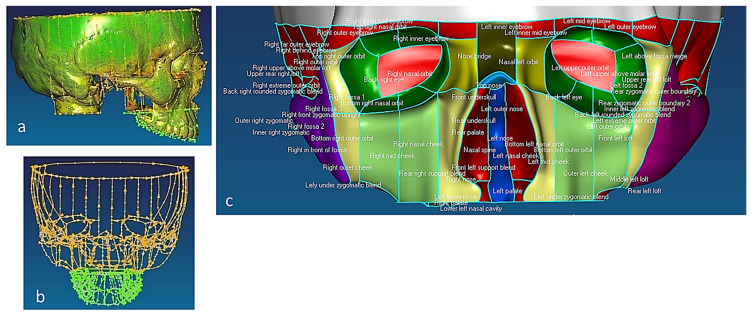
(**a**) Sagittal view of polygonised point clouds, displaying cloud curvature colours. (**b**) Frontal view of maxilla with point clouds and parameterised B-spline curves network, and (**c**) extracted features of facial bones, such as frontal, orbits, nasal, zygomatic, temporal, and cheek [69,171].

Stages 5–11 (see Section 1.2) of the RE framework are shown in Figure 7, Figure 8, Figure 9 and Figure 10 for mandible, dentitions and maxilla, respectively. The Gaussian curvature visualisation helps to aid the bionic design process for successful shape segmentation and feature extraction as shown in Figure 7a,b. Curve/surface modelling and optimisation of the biological structure are shown in Figure 7c–e, Figure 8, Figure 9 and Figure 10.

#### 2.1.3. Phases 4, 5—Mapping Clinical Chewing Trajectory and Computational Validation

**Figure 11:**
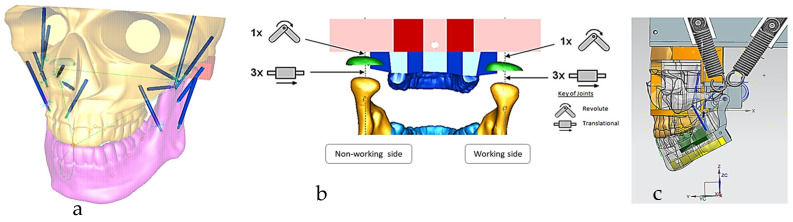
(**a**) Digital skull model with chewing trajectory and mechanical muscle actuators, where muscle insertions and origins were selected on the mandible and maxilla, respectively. (**b**) Boundary conditions and relationship between the maxilla, TMJ disc and mandible, representing 6 DOFs. Constraints and geometrical relationship between the geometric kinematic axis (left/right condylion) and incisor points and chewing trajectory (non-working and working sides) are also shown. (**c**) Sagittal view of the muscle data (7 on each side) and alternate bilateral chewing springs representing the temporalis muscle [69].

The chewing trajectory information was digitised and combined with origin and insertion coordinates (x, y, z) for the lines of action of muscles [172,173]. They were subsequently mapped and constrained to the digital skull model as shown in Figure 11 before carrying out a multi-body dynamic simulation. In this way, the accuracy of biomimetic modelling from phase 3 was verified with chewing functions (articulation, occlusion and mastication).

### 2.2. Hand 3D Data Acquisition, Biomimetic Modelling

The section is split in a similar fashion to Section 2.1; however, the six phases cover slightly different process, which are as follows:

**Phases 1, 2**—Digitisation process and pre-alignment of scanned data (point clouds).

**Phase 3**—Segmentations, features extraction, and curve, surface and solid modelling.

**Phases 4, 5**—Computational evaluation with multi-body dynamics and FEA.

**Phase 6**—Prototyping and proof of concept (will be explained in Section 3.2).

#### 2.2.1. Phases 1 and 2—Digitisation Process and Pre-Alignment of Scanned Data (Point Clouds)

**Figure 12:**
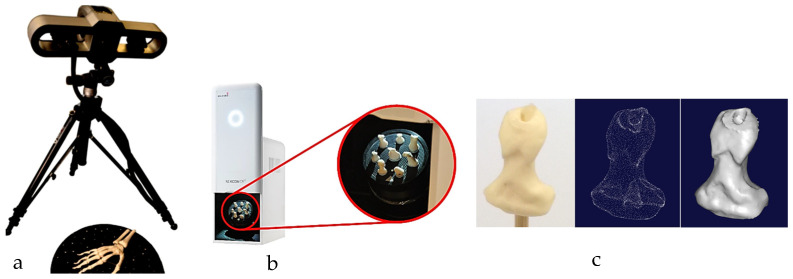
(**a**) Scanning the whole artificial hand using Rexcan 4 with integrated turntable. (**b**) Scanning the 27 individual bones using Rexcan DS2. (**c**) A sample of physical distal phalanx model and scanned data in 3D point clouds and a 3D mesh.

**Figure 13:**
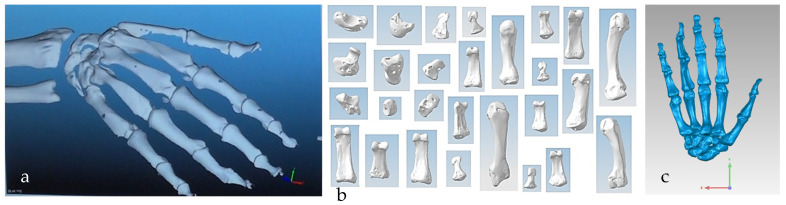
(**a**) Scanned point clouds of the 29 hand bones in Solutionix ezScan™. (**b**) Individual scanned point clouds. (**c**) Pre-alignment digitisation results of 27 bones in Geomagic Wrap^®^.

Combinations of non-contact, 3D white light scanners were used for digitisation processes (see Section 1.5.2), as shown in Figure 12a–c, which shows the physical model of a distal phalanx and the corresponding scanned data as a 3D point cloud and 3D mesh. All 29 bones were then mapped into the global scan and pre-aligned/registered, as shown in Figure 13.

1. **Data cleaning of scanned point clouds.**

**Figure 14:**
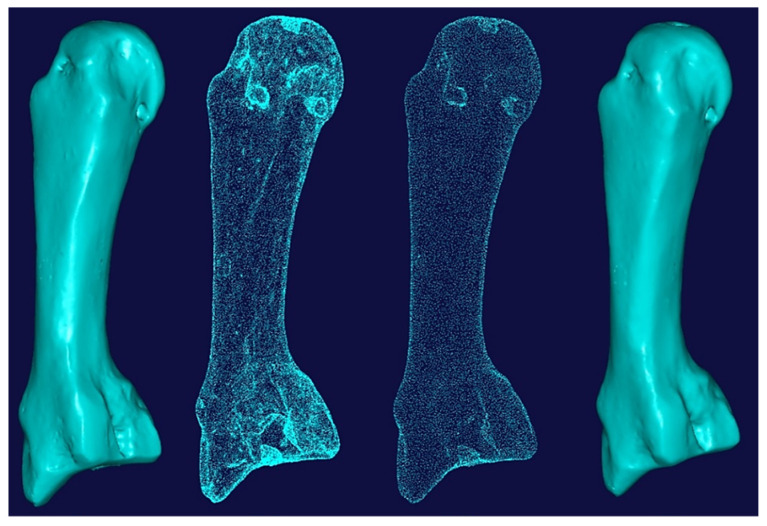
Cleaning and removing noise from the scanned data (point clouds) of a metacarpal bone.

Figure 14 shows the process of cleaning and removing noise from a metacarpal bone’s scanned data and optimising the point cloud for the segmentation process This was repeated for all the other hand bones.

#### 2.2.2. Phase 3—Segmentations, Feature Extraction, and Curve, Surface and Solid Modelling

**Figure 15:**
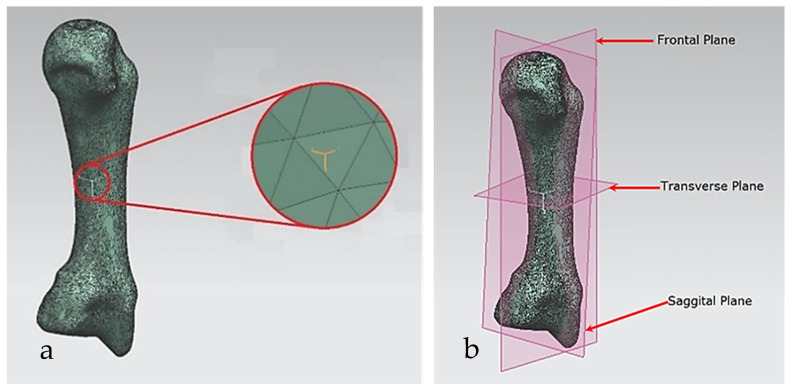
(**a**) CoG and its principal axes of a metacarpal bone. (**b**) A metacarpal’s principal planes.

**Figure 16:**
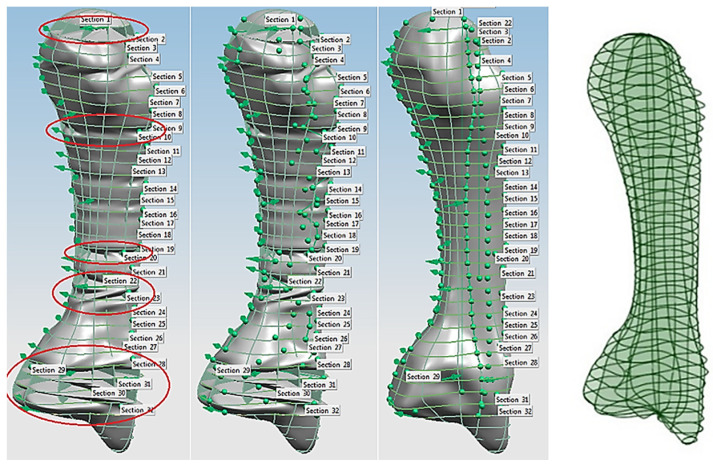
The process of curve network optimisation on a metacarpal bone to obtain nature design intent shape.

**Figure 17:**
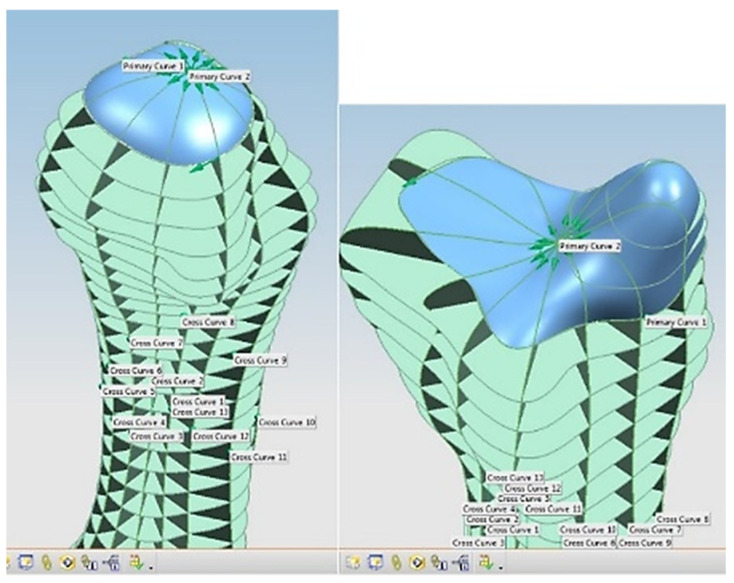
Feature extraction from a metacarpal bone with its head and end features as extracted surfaces.

**Figure 18:**
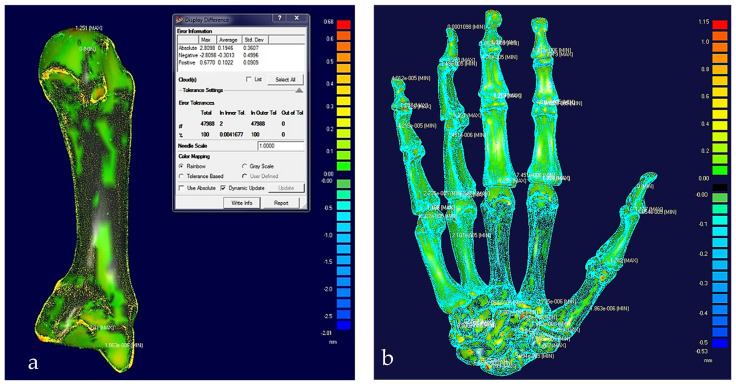
(**a**) Colour difference map on a middle metacarpal bone with a maximum error of 0.68 mm. (**b**) Colour difference map on hand bones with a maximum error of 1.15 mm.

**Figure 19:**
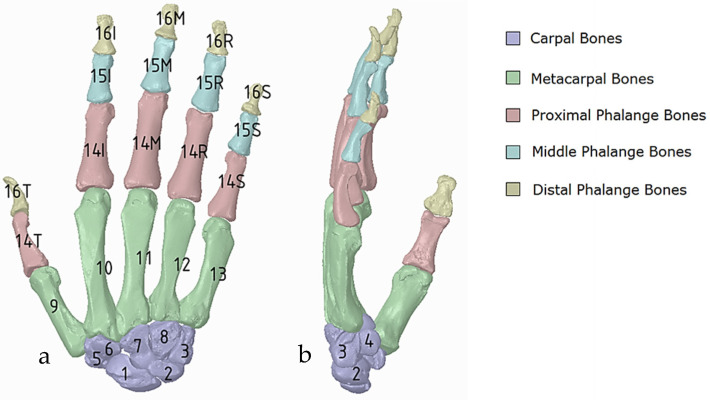
(**a**) Diagram of the five categories of hand bones in dorsal view; carpals (1–8), metacarpals (9–13), proximal phalanges 14 (thumb, index, middle, ring, small), middle phalanges 15 (thumb, index, middle, ring, small), and distal phalanges 16 (thumb, index, middle, ring, small); the carpals group consist of 8 carpals bones: scaphoid, lunate, triquetrum, pisiform, trapezium trapezoid, capitate, and hamate which are numbered from one to eight, respectively, (**b**) Side view showing number 4, the pisiform.

**Figure 20:**
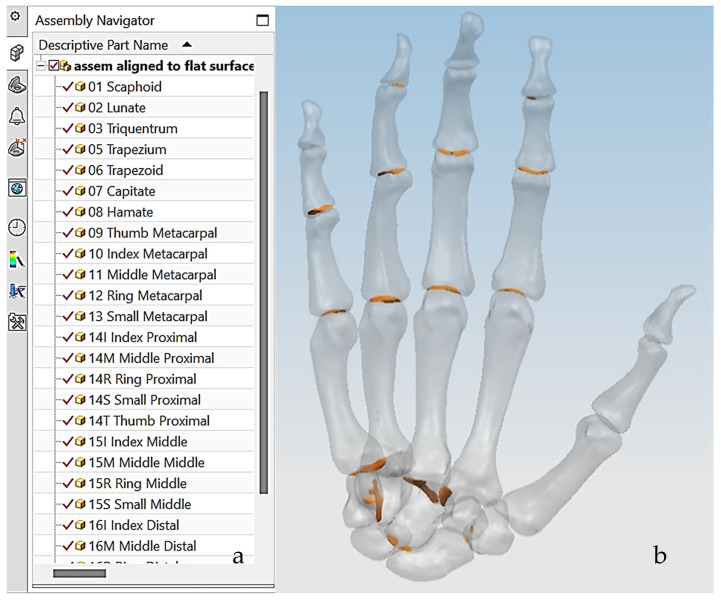
(**a**) Illustration of the hand numbering system mapped into the assembly modelling application of Siemens NX. (**b**) interference (golden colour) existing between the bones in the graphical view.

**Figure 21:**
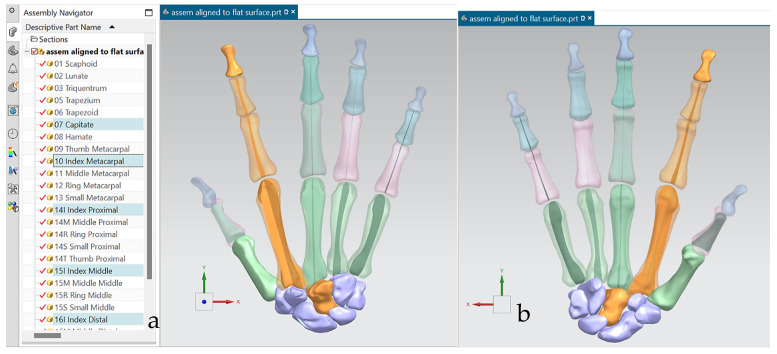
(**a**) Anatomically aligned fingers and carpal in dorsal view, where capitate and index fingers bones are selected according to their numbering system. (**b**) Palmar view of five fingers and selected carpal bones.

This phase was more challenging compared to the previous work illustrated for the skull because of the number of bones and DOF that the biological object consisted of. Hence, the RGEs strategy, segmentation process, and anatomical alignment/registration were far more complex. This section covers stages 5–11 of the RE framework (see Section 1.2) to anatomically align the hand bones and establish the form-function connection. This is illustrated across Figure 15, Figure 16, Figure 17, Figure 18, Figure 19, Figure 20 and Figure 21.

1. **Hand RGEs strategy and segmentation process.**

As implemented in the biomimetic modelling of the skull, the RGEs process was used for identifying the geometrical entities (points, lines, axes, planes) of hand bones and their characteristics such as directions, planes, and views based on anatomical landmarks [174]. Moreover, the novel RGEs method used as a segmentation process obtained the nature design intents, extracting accurate locations of joints, tendon attachment, bone alignment, and also the hand’s pulley system.

For each bone, the process started with identification of the centroid point, which is the bone’s centre of gravity (CoG) and its principal axis as illustrated in Figure 15a,b, respectively. Principal planes were created on a metacarpal bone, as shown in Figure 15b. This process was repeated for the rest of the hand bones, except for the carpal’s 8 bones that were more complex. The strategy presented in Figure 9 and Figure 10 was adapted for this scenario due to their unique structural geometry.

Once this key geometrical information (i.e., CoG and principal planes) was mapped onto the bone 3D mesh (i.e., point clouds), radial and transverse cross-sections were used as a segmentation technique to partition the bone. Subsequently, a B-spline curve network was created for feature extraction and both surface and solid modelling. Figure 16 shows the optimisation process to obtain the geometric design intentions.

By grouping the hand bones according to their shapes, feature extraction for each bone in the phalanges and metacarpal groups was classified as head, end and base features as shown in Figure 17.

The final stage of creating a metacarpal bone surface/solid model, was uniting the three features (i.e., head, base and end) together. The whole process was repeated for each of hand bone to complete surface and solid modelling.

2. **Verify the integrity of pre-aligned hand bones solid model.**

After the modelling processes, the solid models of the hand’s bones (and their tolerances) were verified, with each scan optimised corresponding to bone point clouds. Figure 18 shows the colour difference maps for a middle metacarpal bone and the overall structure of the hand bones.

3. **Alignment and functional registration according to RGEs strategy.**

This work utilised an assembly modelling technique (i.e., top-down design; see Section 1.3) to support a geometric, feature-based modelling approach (i.e., bottom-up design) and RGEs strategy to virtually assemble the hand. Therefore, the numbering system was adapted with their medical terminology, as shown in Figure 19.

4. **Numbering Individual Digital Hand Bone**

Figure 20a shows the implantation of the hand numbering system into the assembly modelling application of Siemens NX. Figure 20b shows the interference analysis on the bone assembly revealed that there was some interference between contacting bones that existed within the pre-alignment scan data.

The bones were realigned according to their principal planes RGEs strategy (see Section 1.5.2) to make sure that the rotation of the fingers (i.e., function) was in the correct orientation. The re-alignment of metacarpal and carpal bones was more complex due to their complex shape and the absence of a principal reference axis.

Figure 21 shows the five fingers with their corresponding numbering system after being anatomically aligned [175] in dorsal and palmar views, respectively.

#### 2.2.3. Phases 4, 5—Computational Evaluation with Multi-Body Dynamics and FEA

In this study, a torque-driven model based on postural synergies (see Section 1.5.4) was applied in stage 3 of the RE framework to assess the behaviour of digitally assembled hand bones. This aimed to evaluate stages 1 and 2 for dynamic models (see Section 2.2.2) design intentions, correct physiological alignment of the hand, and the forces required to move fingers for completing different tasks. This was the most critical knowledge abstraction phase for an anthropometric tendon-driven prosthetic hand before designing the joint mechanism and prototyping the hand for stage 4.

##### Siemens NX Multi-Body Dynamic (MBD) Simulation and FEA

1. **Identification of centre of joints.**

**Figure 22:**
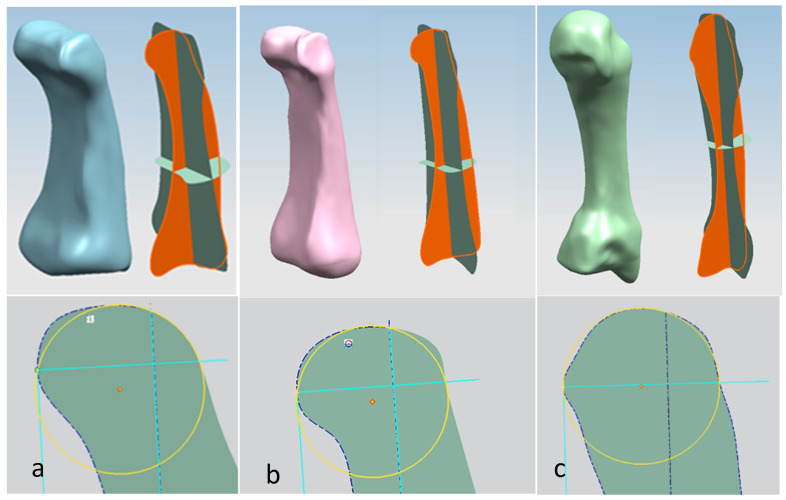
Illustration of the RGEs process for creating COR on head shapes using cross-sectional principal planes. (**a**) Middle phalanx. (**b**) Proximal phalanx. (**c**) Metacarpal bone.

An example of feature extraction for the head of a metacarpal bone was illustrated in phase 3 of the RE process (Figure 17). The cross-sectional shapes of the head features were used to identify the CORs for the metacarpal bones, proximal phalanges and middle phalanges as shown in Figure 22.

**Figure 23:**
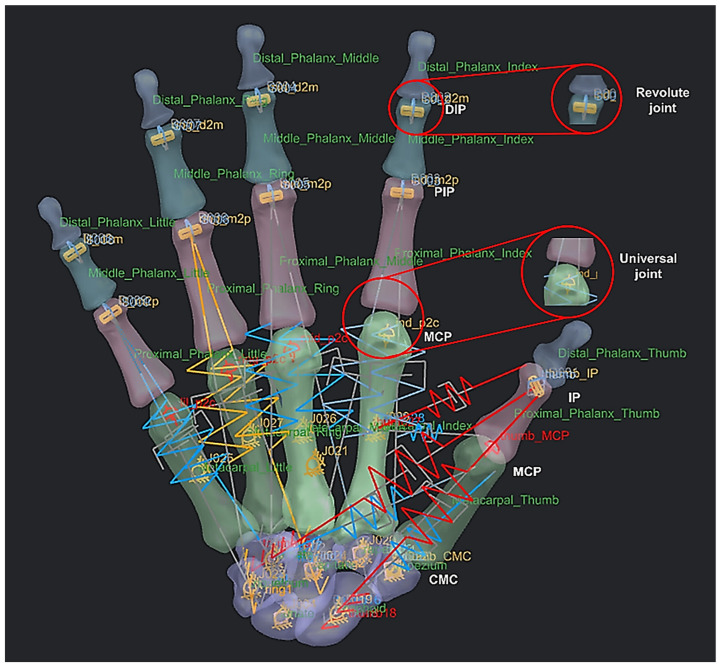
An example of a spring-damper system setup (instead of tendons and muscles) as linear and rotational springs with 27 bones as rigid bodies or links, 9 revolute joints for the distal interphalangeal and proximal interphalangeal, and 6 universal joints for the metacarpal phalangeal and carpometacarpal.

Figure 23 shows an example of a spring-damper system that was setup and modelled for linear and rotational springs with their respective properties that includes stiffness, damping, dimensions, and pre-load force. This process was repeated to identify the rest of the CORs, except for the carpal bones. The behaviour of the hand also included 4 DoFs in each finger and 5 DoFs in thumb.

2. **Postural synergies simulation and clinical validation.**

**Figure 24:**
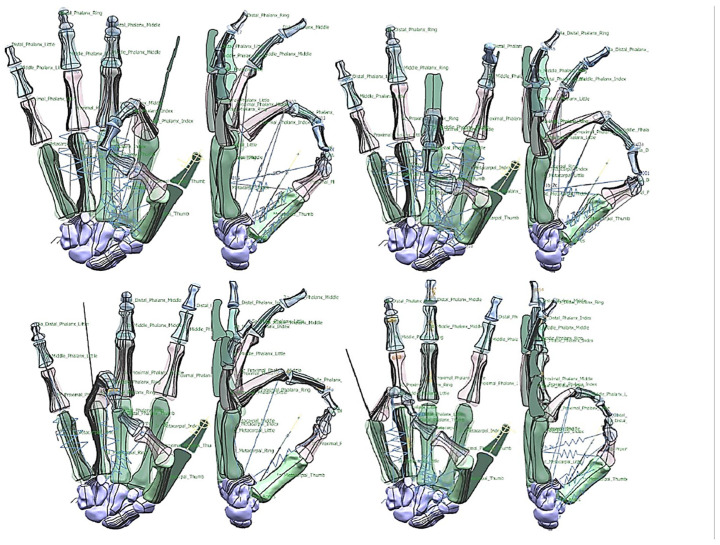
Diagrams of the pinch simulation for every finger.

**Figure 25:**
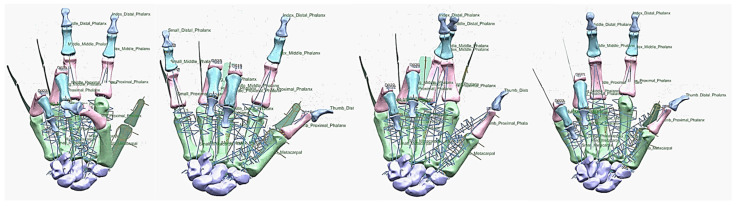
Different hand poses (the peace sign, rock sign, crossed finger. and three sign). The peace sign has similar characteristics with ring pinch grip with small finger in flexion. The rock sign and three sign shared similar characteristics with different finger in flexion.

**Figure 26:**
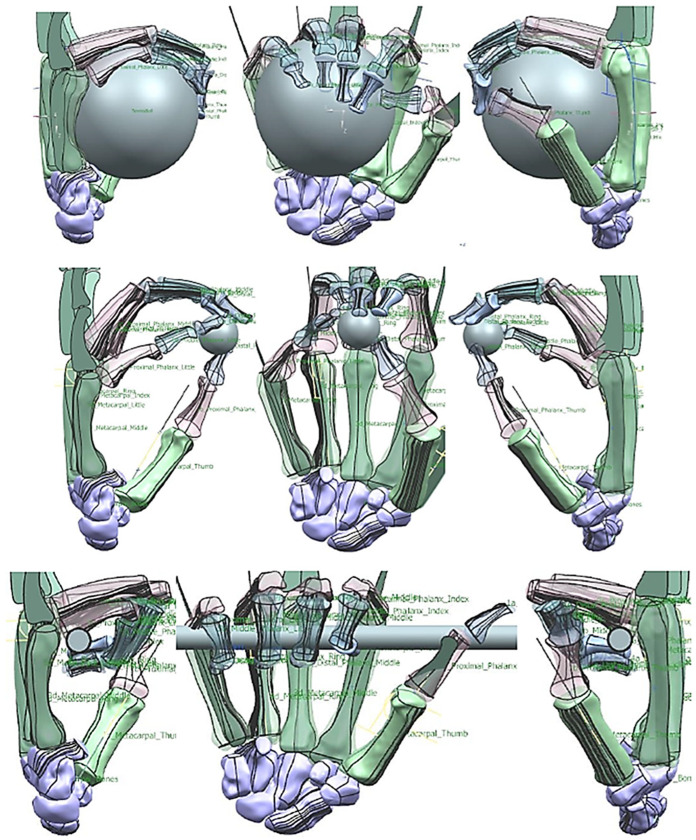
Diagrams of the grasping ability in different views.

**Figure 27:**
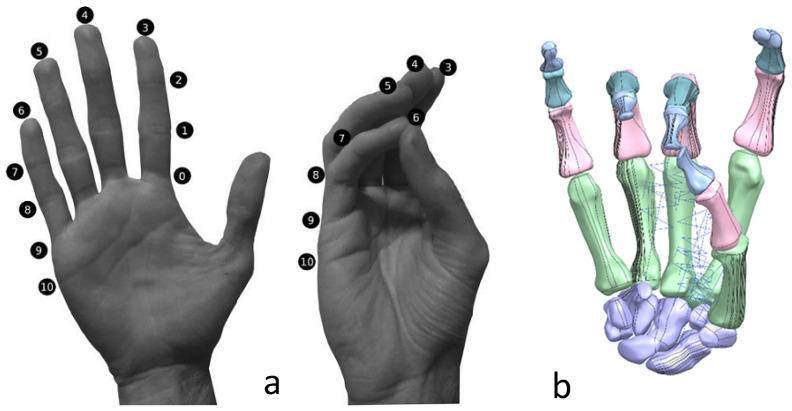
(**a**) Kapandji 0–10 test positions or locations [170]. (**b**) Simulated Kapandji position 4 (middle pinch).

**Figure 28:**
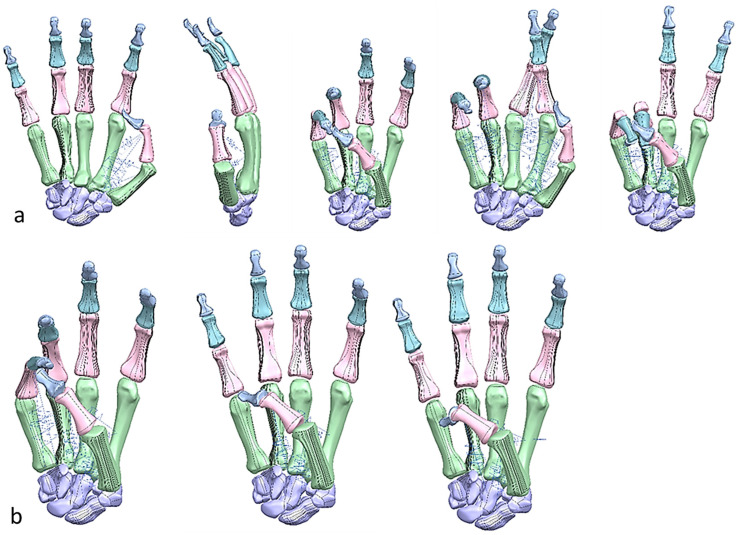
(**a**) Simulated Kapandji positions 0 and 6 (crossed fingers and 2nd dactylonomy gestures). (**b**) Simulated Kapandji positions 8–10.

Torque-driven simulation of various poses and daily activities based on postural synergies were simulated and analysed for simple and complex motion. This aided in the design’s optimisation process for clinical alignment and registration of the hand bones before prototyping the tendon-driven artificial hand [167]. Furthermore, the hand’s postural synergies were also simulated [176,177] and analysed for grasping ability of objects that varied in size and shape [156] with the added benefit of adapting two clinical assessments tests (the Fugl-Meyer assessment (FMA) [178,179], and Kapandji test [180,181,182]) for evaluating the design method. These two clinical assessments are commonly used to assess hand function in post-stroke patients. This provided assurance that the biological knowledge abstraction and the design intentions captured through the RE framework were comparable with clinical assessment of human hands. The contact reaction forces for a spherical grip were calculated using the total forces exerted by a maximum grip strength of a male (between 400 N and 600 N) [183,184,185,186]. Figure 24, Figure 25, Figure 26, Figure 27 and Figure 28 show the details of the postural synergies’ simulation, analysis, and validations.

3. **Hand alignment evaluation.**
  1. **Pinch and posing.**
  2. **Grasping various objects.**
  3. **Clinical validation with Fugl-Meyer assessment (FMA) and Kapandji test.**

The spring-damper systems were setup for pinch, pose, or grasp positions and optimised according to FMA and Kapandji test [170] procedures to control the movements of hand bones. The primary factors to successful clinical validation were understanding the spring stiffness, the damping coefficient, and the preloaded length or angle for translational or rotational spring dampers. In addition, relating the spring-damper systems to torque-driven models was key to characterise all the muscles acting across a joint.

FMA feature assessments were based on taxonomy conducted by Controzzi and co-workers [177] for assessing the postural synergies that identified and classified difference grasps including hook grasp, thumb adduction, pincer, cylindrical, and spherical grasps. Examples of a simulated model of the Kapandji in a different positions are shown in Figure 27 and Figure 28.

## 3. Results—Innovative, Bio-Inspired Product Development

### 3.1. Phase 6—Prototyping, Proof of Concept and Clinical Validation of a Drug-Releasing Chewing Robot

**Figure 29:**
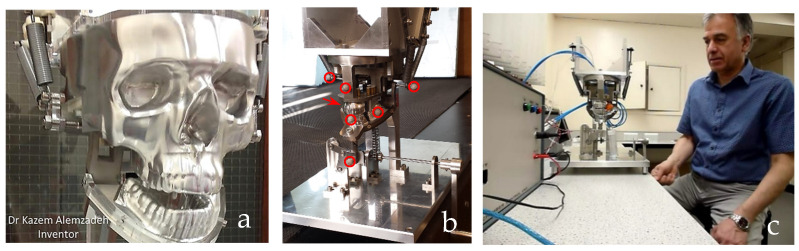
(**a**) Fully functional biomimetic humanoid chewing robot prototype. (**b**) Inverse-kinematic motion analysis using 7 optical markers (highlighted red) for optimising mechanics of chewing, mechanical occlusion, chewing motion and chewing cycle duration (1 chew per second), and verifying 4 bar linkage mechanism chewing trajectory before clinical evaluation. (**c**) Process of clinical validation with built-in artificial oral environment [69].

Developing masticatory apparatus with an artificial oral environment is of interest to (i) food science, focusing on bolus breakdown with flavour release; (ii) dental science, for material testing and failure points; and (iii) the pharmaceutical industry, for drug release. However, apparatus that closely mimics human chewing and oral conditions has yet to be realised [69]. Figure 29 is related to drug release from medicated chewing gum (MCG) that has been recognised as a new and advanced delivery method with a promising future. Its potential has not yet been fully exploited, because currently there is no gold standard for testing the release of drugs from chewing gum in vitro [69]. Figure 29 shows a novel humanoid chewing robot created and clinically validated by Alemzadeh and co-workers [69]. It is capable of closely replicating the human chewing motion in a closed environment, incorporating artificial saliva and allowing measurement of xylitol release from the gum. The release of xylitol from commercially available chewing gum was quantified following both in vitro and in vivo mastication. The chewing robot demonstrated a similar release rate of xylitol as human participants. The greatest release of xylitol occurred during the first 5 min of chewing, and after 20 min of chewing, only a low amount of xylitol remained in the gum bolus, irrespective of the chewing method used. Saliva and artificial saliva solutions, respectively, were collected after 5, 10, 15, and 20 min of continuous chewing and the amount of xylitol released from the chewing gum determined. These results demonstrate that the chewing robot with built-in humanoid jaws could provide opportunities for pharmaceutical companies to investigate and refine drug release from gum, with reduced patient exposure and costs [69].

This innovation required an interdisciplinary team combining the fields of bionics, bio-engineering, dental biomechanics, biomedical engineering, and participant studies. This invention was validated by in vitro/in vivo comparison of drug release rates to achieve local therapy from the controlled release of API from MCG. This interdisciplinary research was conducted by two internationally recognised research groups from the Engineering Systems, Design and Innovation in the School of Electrical, Electronic and Mechanical Engineering, and the dental Clinical Trials Unit within the Bristol Dental School from the Faculty of Engineering and Health Science at the University of Bristol. This brought together two groups with very different knowledge and research method backgrounds.

### 3.2. Prosthetic Hand—Analysis of Spherical Grip Contact Reaction Forces

#### Phase 6—Prototyping and Proof of Concept of an Anthropometric Prosthetic Hand

1. **Hand actuation design.**

**Figure 30:**
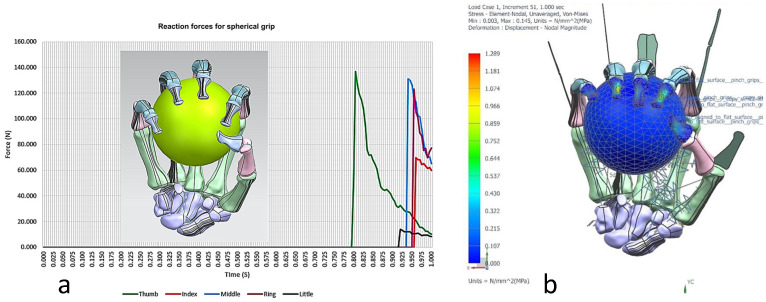
(**a**) Spherical grip contact reaction forces for the simulated hand grasping a tennis ball (67 mm). The maximum force exerted was at the thumb with 137 N and total force for the hand was 475 N. (**b**) FEA of the same setup showing von Mises stresses with associated deformation.

**Figure 31:**
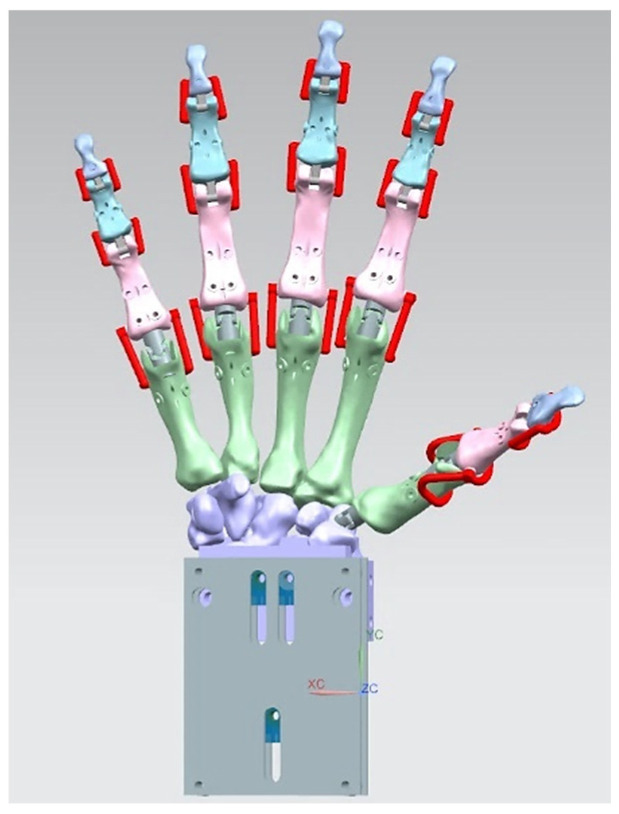
Passive extensors (red colour) and the overall design of the underactuated system, which consists of single structure of carpal bones. Part of carpal bones were modified to suit the requirements for an underactuated design [187].

Integrated 3D motion analysis and finite element analysis (FEA) conducted as a computational evaluation method for contact force calculations and maximum grip strength to validate the hand as shown in Figure 30. A differential pulleys single actuation mechanism was designed to manually actuate the artificial hand as shown in Figure 31. The motion of the wrist is related to the carpal bones, which were considered as fixed and designed as a single structure. Figure 31 shows the underactuated artificial hand design that consists of all fingers with their joints and passive extensors, single structure carpal bones, and a tendon-driven mechanism. Flexion tendons and the passive extensor were designed as a proof of concept for the tendon-driven application.

Passive extensors (red colours) were designed with Ninja semi-flex material to improve the extension motion of the fingers by reducing the complexity of the tendon routing tremendously when compared with active dorsal extensor tendons [187].

2. **Prototyping and concept proof.**

**Figure 32:**
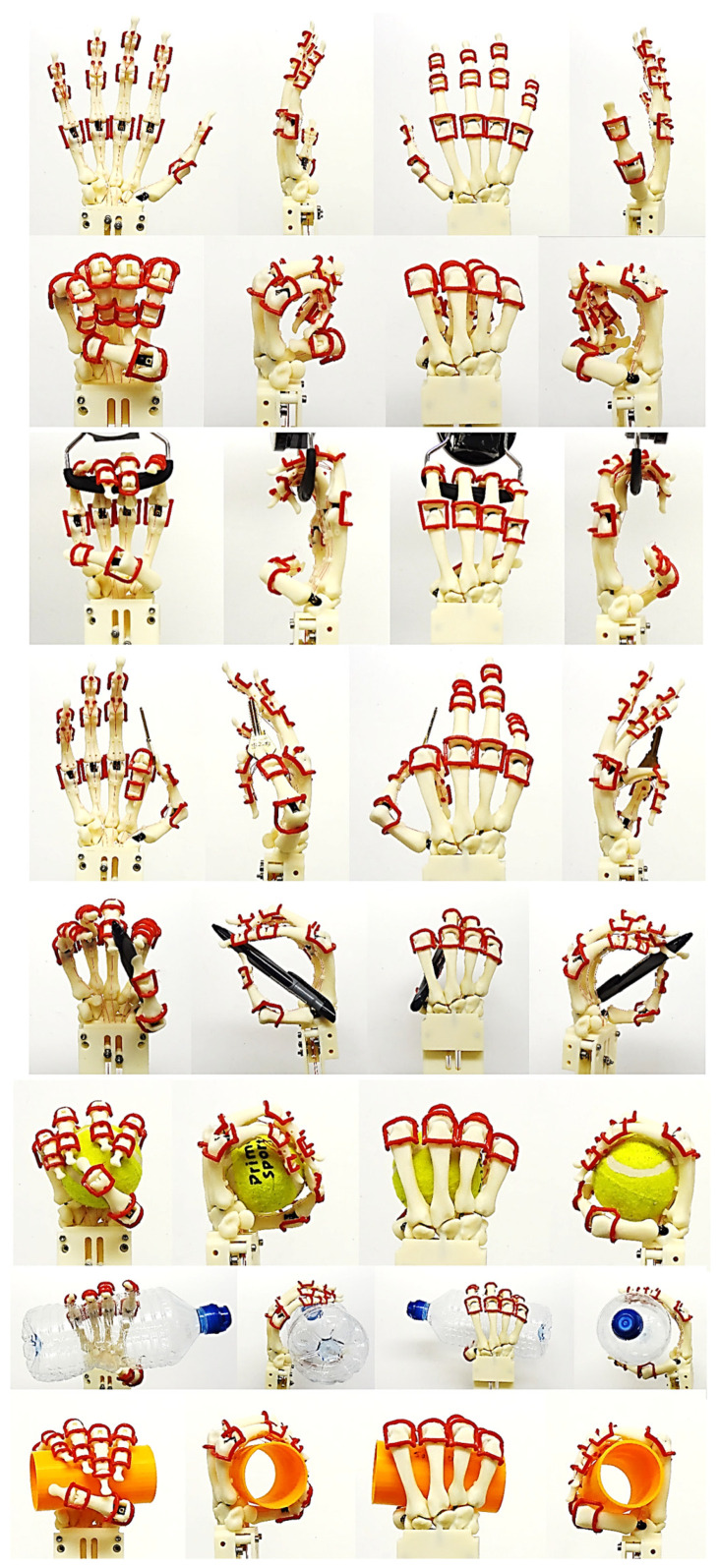
Different views of seven prosthetic hand positions: (from top to bottom) full extension, full flexion, hook grip, key grip, tripod grip pencil, spherical grip, and cylindrical grips (water bottle and 50 mm diameter cylinder). The prosthetic hand actuated manually by differential pulleys single actuation mechanism showing the grasping performance of 21-DoF capability [187].

The prosthetic hand protype was assembled with its actuation mechanism and assessed with all seven positions of the Fugl-Meyer assessment (FMA) [178,179]. These positions are shown in Figure 32.

## 4. Discussion

**Figure 33:**
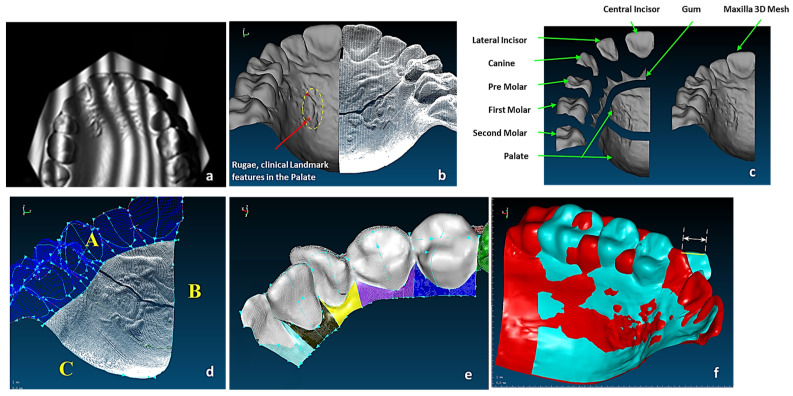
(**a**) Scan data with digital fringe projection of a dental cast model [189]. (**b**) Comparison of the 3D polygon mesh with the original point cloud data [189]. (**c**) Maxilla segmentations process (i.e., feature extraction) with their associated clinical names. (**d**) Boundary curves A, B and C for palate. (**e**) Gum surfaces after merging. (**f**) Superimposed 3D CAD models of pre-treatment (red) and post-treatment (blue) models, allowing a linear measurement (of 4.931 mm) to be made to change in canine position with treatment [190].

Phases 1–3 of skull presented in Section 2.1 of this study have been already applied to clinical dentistry. This was the use of virtual 3D images to aid diagnosis in treatment planning and appliance, instead of the use of impression materials together with plaster or stone models. Figure 33 shows the 3D measurement of virtual teeth movements during the pre-post orthodontic treatment. The Rugae, which are clinical landmarks in the Palate (located at the roof of the maxilla) was used as a basis for stable reference points [188] during clinical alignment or registration.

The goal of the novel reverse biomimetic approach was implementation of functional feature extraction, surface reconstruction, and solid modelling into five stages of a design process. Additionally, virtually mapping this in a pictorial fashion with high-end software fosters simpler understanding and representation of knowledge transfer from biology to engineering, and can lead to innovative bio-inspired development.

In the author’s view as an academic, introducing bio-inspired product development into engineering courses and implementing them into undergraduate courses [191,192,193] would be one method to bolster interest and encourage growth within the subject area. As a matter of fact, extensive research was conducted between 2009 and 2021 [194,195,196,197,198,199] by a number of researchers, promoting “A new approach of innovative design: An introduction to C-K theory” [200,201,202]. Design engineering researchers attempted to uncover the logic of the creative process in design engineering with a structure of two spaces, and developed a new theory called Concept-Knowledge (C-K) theory [200,201,202]. The author strongly supports such a notion, and it is hoped that the manuscript contribution would be beneficial toward this aim.

## 5. Conclusions

The manuscript presented a pictorial PLM framework that used bionics as an enabling methodology to bridge the gap between biology and engineering combine life sciences with engineering sciences. The innovative methodology addressed abstraction issues in two phase, double symmetrical abstraction-specification cycles and clearly demonstrated how the abstraction level can be changed from high to low. Abstraction progression into reality was illustrated with two examples of an innovative drug release chewing robot and anthropometric tendon-driven prosthetic hand.

Moreover, the reverse biomimetics approach consisted of eleven technical RE stages for geometrical shape abstraction for ease of knowledge transfer based on functional feature-based modelling (i.e., bottom-up design) and assembling modelling (i.e., top-down design) approaches. The study focused on complex free-form modelling and feature extraction of biological structures from measured point clouds. The novel methods were clearly demonstrated with hybrid geometrical shape abstraction method indicators to characterise surface shapes. Above all, the use of Siemens NX PLM environment create extremely accurate, virtual CAD models based on form-function connections and change the level of abstraction to achieve nature design intents.

## 6. Patents

Dental simulator—Kazem Alemzadeh https://patents.google.com/patent/US20090035739A1/en (accessed 22 December 2024).

## Figures and Tables

**Table 1:** Fayemi’s 8-step process model, known as the “double symmetrical abstraction-specification cycle”.

The Fayemi’s 8-step process model known as “double symmetrical abstraction-specification cycle”	
Problem analysis (1)	Selection of a biological model of interest (5)
Abstraction of technical problem (2)	Abstraction of biological strategies (6)
Transposition to biology (3)	Transposition to technology (7)
Identification of biological models (4)	Implementation and testing in the initial context (8).

**Table 2:** HK—shape classification method illustrating eight surface shapes with their designated colours, condition (K = 0) classified as developable surface with Gaussian curvature is zero. The other conditions are classified as non-developable surfaces with non-zero Gaussian curvature.

		Gaussian Curvature, $K\;=\;{(k}_{1}\;\times \;k_{2}$)		
		K > 0	K = 0	K < 0
**Mean Curvature,** H **=** $\frac{1}{2}$ **(** $k_{1}\;+\;k_{2}$**)**	**H < 0**	Convex (Elliptical or Spherical) 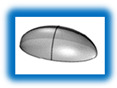 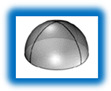	Convex Cylinder 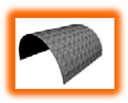	Saddle Ridge 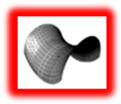
	**H = 0**	(Black colour)	Planar (White colour) 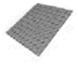	Saddle—Symmetry 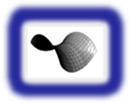
	**H > 0**	Concave (Elliptical or Spherical) 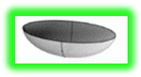 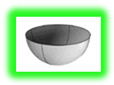	Concave Cylinder 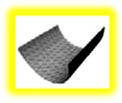	Saddle Valley 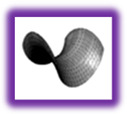

**Table 3:** Shape index (S), illustrating nine surface shapes. S is a number in the range [−1, 1], which covers all shapes (except for planar) with its associated colour map. Conditions (S = (0, ±0.5, ±1)) are critical points (CPs) when shape classification changes in the segmentation process. Curvedness, C, is a positive number that specifies the amount or intensity of surface curvatures; condition C = 0 has no curvedness, and it is flat regions and S unspecified.

$S\;=\;-\frac{2}{\pi }{tan}^{-1}\left(\frac{K1\;+\;K2}{K1\;-\;K2}\right),\;K1\;\geq \;K2$ 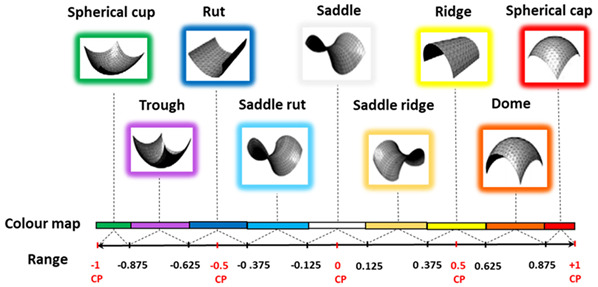 **Shape Index (S)**
$C\;=\;\sqrt{\frac{{K_{1}}^{2}\;+\;{K_{2}}^{2}}{2}}$ 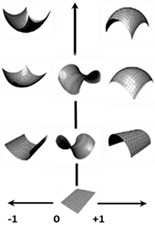 **Curvedness (C)**

## Data Availability

Data are unavailable due to privacy.
